# Direct Conversion of Methane to Ethylene and Acetylene over an Iron-Based Metal–Organic Framework

**DOI:** 10.1021/jacs.3c03935

**Published:** 2023-09-18

**Authors:** Yujie Ma, Xue Han, Shaojun Xu, Zhe Li, Wanpeng Lu, Bing An, Daniel Lee, Sarayute Chansai, Alena M. Sheveleva, Zi Wang, Yinlin Chen, Jiangnan Li, Weiyao Li, Rongsheng Cai, Ivan da Silva, Yongqiang Cheng, Luke L. Daemen, Floriana Tuna, Eric J. L. McInnes, Lewis Hughes, Pascal Manuel, Anibal J. Ramirez-Cuesta, Sarah J. Haigh, Christopher Hardacre, Martin Schröder, Sihai Yang

**Affiliations:** † Department of Chemistry, University of Manchester, Manchester M13 9PL, U.K.; ‡ Department of Chemical Engineering, University of Manchester, Manchester M13 9PL, U.K.; § The Francis Crick Institute, London NW1 1AT, U.K.; ∥ Department of Chemistry, King’s College London, London WC2R 2LS, U.K.; ⊥ Photon Science Institute, University of Manchester, Manchester M13 9PL, U.K.; # Department of Materials, University of Manchester, Manchester M13 9PL, U.K.; ¶ ISIS Facility, Science and Technology Facilities Council, Rutherford Appleton Laboratory, Chilton OX11 0QX, U.K.; ∇ Neutron Scattering Division, Neutron Sciences Directorate, Oak Ridge National Laboratory, Oak Ridge, Tennessee 37831, United States.; ○ Department of Earth and Environmental Sciences, University of Manchester, Manchester M13 9PL, U.K.; ⧫ College of Chemistry and Molecular Engineering, Beijing National Laboratory for Molecular Sciences, Peking University, Beijing 100871, China; ⊗ College of Chemistry, Beijing Normal University, Beijing 100875, China

## Abstract

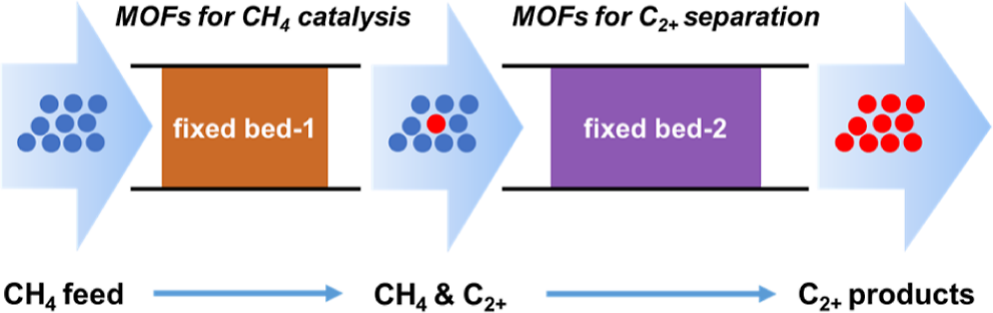

Conversion of methane (CH_4_) to ethylene (C_2_H_4_) and/or acetylene (C_2_H_2_) enables routes to a wide range of products directly from natural gas. However, high reaction temperatures and pressures are often required to activate and convert CH_4_ controllably, and separating C_2+_ products from unreacted CH_4_ can be challenging. Here, we report the direct conversion of CH_4_ to C_2_H_4_ and C_2_H_2_ driven by non-thermal plasma under ambient (25 °C and 1 atm) and flow conditions over a metal–organic framework material, MFM-300(Fe). The selectivity for the formation of C_2_H_4_ and C_2_H_2_ reaches 96% with a high time yield of 334 μmol g_cat_^–1^ h^–1^. At a conversion of 10%, the selectivity to C_2+_ hydrocarbons and time yield exceed 98% and 2056 μmol g_cat_^–1^ h^–1^, respectively, representing a new benchmark for conversion of CH_4_. In situ neutron powder diffraction, inelastic neutron scattering and solid-state nuclear magnetic resonance, electron paramagnetic resonance (EPR), and diffuse reflectance infrared Fourier transform spectroscopies, coupled with modeling studies, reveal the crucial role of Fe–O(H)–Fe sites in activating CH_4_ and stabilizing reaction intermediates via the formation of an Fe–O(CH_3_)–Fe adduct. In addition, a cascade fixed-bed system has been developed to achieve online separation of C_2_H_4_ and C_2_H_2_ from unreacted CH_4_ for direct use. Integrating the processes of CH_4_ activation, conversion, and product separation within one system opens a new avenue for natural gas utility, bridging the gap between fundamental studies and practical applications in this area.

## Introduction

Direct conversion of methane (CH_4_) to C_2+_ unsaturated hydrocarbons under mild conditions is an important chemical process.^1−3^ However, this reaction remains a major challenge in both industry and academia due to the significant thermodynamic barrier under practical conditions. The current industrial route is indirect with CH_4_ converted to syngas, a mixture of CO and H_2_, which affords liquid fuels via the Fischer–Tropsch process at high temperatures and pressures.^4,5^ There are thus powerful drivers to develop more efficient catalysts that enable direct conversion of CH_4_ to commodity chemicals.^6−12^ A key step in controlling the reactivity of CH_4_ is the activation of the C–H bond and stabilization of key reaction intermediates over efficient active sites to suppress complete dehydrogenation and over-oxidation to CO_2_. The material Fe@SiO_2_ shows exceptional catalytic activity for direct conversion of CH_4_ to C_2_H_4_, aromatics, and H_2_ at 1090 °C.^13^ Emerging photo-catalytic loop and flow systems^9−11^ have been reported to promote the conversion of CH_4_ to C_2_ and C_3_ hydrocarbons under ambient conditions, but these systems rely heavily on the use of noble metal catalysts and productivities remain low (typically < 100 μmol g_cat_^–1^ h^–1^). Non-thermal plasma (NTP) is widely recognized as a promising technique that can enable thermodynamically challenging reactions.^14−16^ The highly energetic electrons generated in NTP can activate inert molecules such as CH_4_ under mild conditions (room temperatures and ambient pressure).^16^ Plasma-activated CH_4_ dimerization systems have also been developed.^17,18^ However, these often suffer from low selectivity for unsaturated C_2+_ hydrocarbon products, and the reaction mechanisms are poorly understood. The precise identification of the active sites within catalysts and elucidation of their roles in catalytic CH_4_ conversion remain elusive due to the lack of direct structural information and limited methods of characterization. Although significant advances have been made in this research area over the past few decades, state-of-the-art CH_4_ catalysis systems typically afford CH_4_ conversion of <10%. Therefore, the separation of products from large amounts of unreacted CH_4_ is a further challenge that needs to be overcome for practical applications. To date, a catalytic system combining the advantages of high CH_4_ conversion and high selectivity for unsaturated C_2+_ products, operating at ambient conditions, with ease of product separation from unreacted CH_4_, and detailed clarification of the role of a catalyst in the activation of CH_4_ has not been achieved.

**Figure 1:**
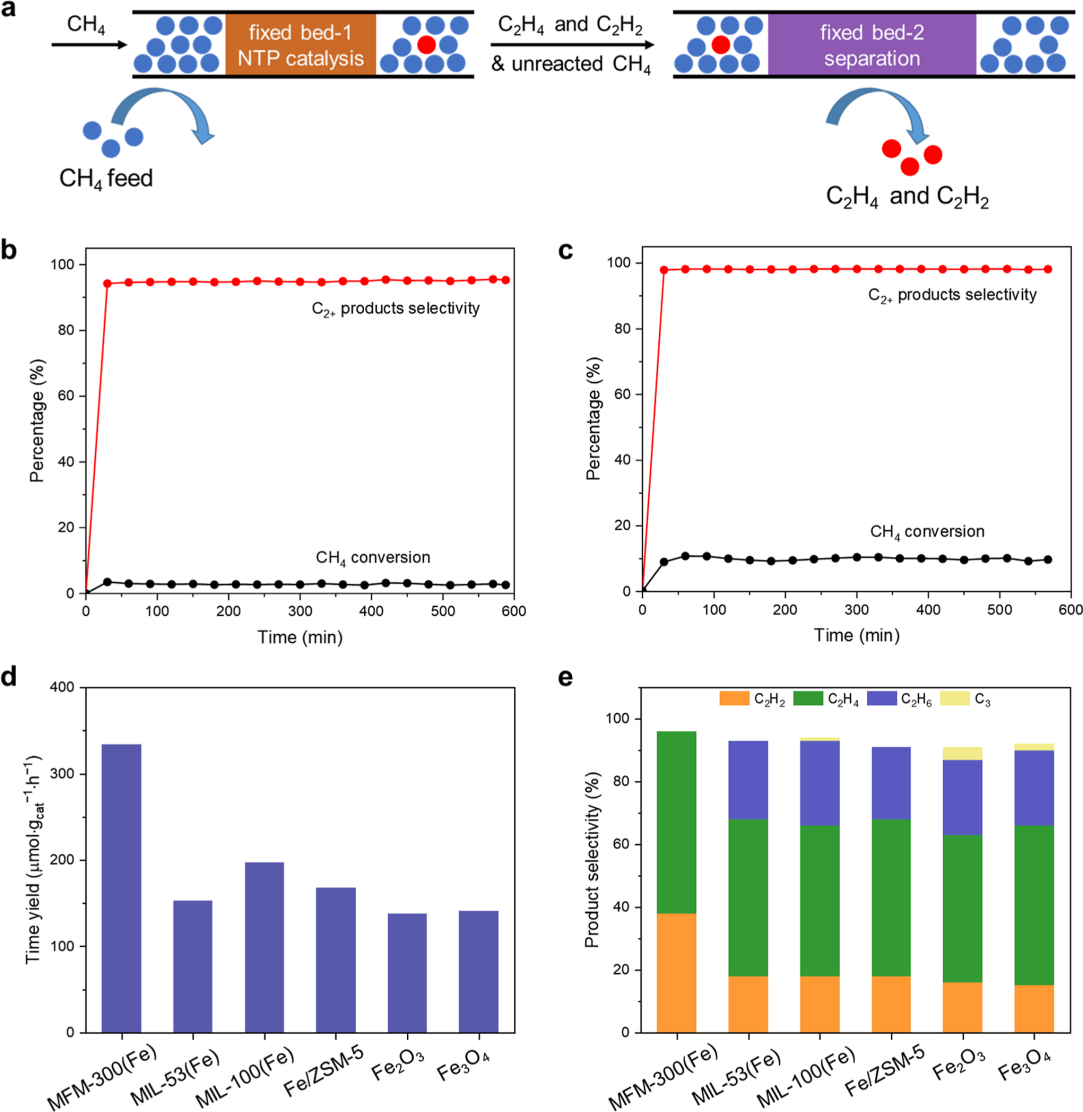
(a) Cascade system composed of two fixed-beds. Fixed-bed-1 is packed with catalyst for conversion of CH_4_. Fixed-bed-2 is packed with porous materials (HKUST-1 or ZSM-5) for separation of C_2+_ products from unreacted CH_4_. (b,c) Time-on-stream (ToS) plots of the catalytic performance over MFM-300(Fe) under steady-state NTP conditions at a specific energy input of 2 kJ L^–1^ for (b) and 8 kJ L^–1^ for (c). Reaction conditions: 1 and 2% CH_4_ in He for (b) and (c), respectively, with a total flow rate of 60 mL min^–1^ for the feed gas; 60 mg of catalysts at 25 °C, and 1 atm. (d,e) Comparison of the catalytic activity (time yield for total C_2+_ products) and product selectivity over different catalysts (specific energy input of 2 kJ L^–1^, 1% CH_4_ in He as feed gas).

Metal–organic framework (MOF) materials are highly designable with diverse active sites and well-defined structures, providing an excellent platform for the control and detailed analysis of their role in catalysis.^19−21^ Here, we report a cascade system based upon MOFs to integrate the direct conversion of CH_4_ to C_2_H_4_ and C_2_H_2_ and the efficient separation of C_2+_ products from unreacted CH_4_. Under the activation of NTP and ambient flow conditions (25 °C and 1 atm), MFM-300(Fe) (MFM = Manchester Framework Material) binds and activates CH_4_ to form CH_*x*_ species which dimerize to yield C_2_H_4_ and C_2_H_2_. A superior time yield of 334 μmol g_cat_^–1^ h^–1^ and a high selectivity of 96% to C_2_H_4_ and C_2_H_2_ is achieved at a CH_4_ conversion of 3%. At a higher CH_4_ conversion of 10%, the total selectivity for C_2+_ hydrocarbons reaches 98% with an exceptional time yield of 2056 μmol g_cat_^–1^ h^–1^. We find that the adsorption of CH_4_ molecules to the Fe–O(H)–Fe sites within MFM-300(Fe) is crucial to the activation of the C–H bond, as confirmed by CH_4_ isotherms, in situ neutron powder diffraction (NPD), inelastic neutron scattering (INS) and density functional theory (DFT) calculations. In situ diffuse reflectance infrared Fourier transform (DRIFT), solid-state nuclear magnetic resonance (ssNMR) and electron paramagnetic resonance (EPR) spectroscopies reveal the critical role of the Fe–O(H)–Fe sites in stabilizing the reaction intermediates, forming an Fe–O(CH_3_)–Fe adduct and enabling a highly efficient and selective CH_4_-to-C_2+_ process. Complementary experimental and modeling techniques thus provide valuable insights into the future design and development of new efficient and selective catalysts for activation of CH_4_. In addition, a secondary fixed-bed packed with benchmark MOF/zeolite materials (Figures 1a and S1) achieves online separation of C_2_H_4_ and C_2_H_2_ from unreacted CH_4_ in a cascade system. This work demonstrates the first example of a cascade system that enables the efficient and selective activation and conversion of CH_4_ and product separation in one system under ambient and flow conditions.

## Results and Discussion

### Synthesis and Characterization

MFM-300(Fe) exhibits an open framework structure comprised of *cis*-[Fe(OH)_2_O_4_]_∞_ chains bridged by tetracarboxylate linkers.^22,23^ The crystallinity, porosity, and morphology of the as-synthesized MFM-300(Fe) were analyzed by powder X-ray diffraction (PXRD, Figure S2), N_2_ adsorption isotherm (Figure S3 and Table S1), scanning electron microscopy (SEM, Figure S4), and high-angle annular dark-field scanning transmission electron microscopy (HAADF-STEM, Figure S5), demonstrating the successful preparation of this material. The presence of Fe–O(H)–Fe sites in MFM-300(Fe) was confirmed by NPD (Figure S6) and FTIR spectroscopy (Figure S7). The trivalent oxidation state of the Fe(III) centers was confirmed by X-ray absorption near-edge structure (XANES, Figure S8) and X-ray photoelectron spectroscopy (XPS, Figure S9). Analysis of the extended X-ray absorption fine structure (EXAFS) data of MFM-300(Fe) (Figure S8) fits well for an Fe–O path of ∼2.00 Å. The structures of bare MFM-300(Fe) and gas-loaded materials have been determined by NPD (Figures S10–S13).

### Studies of Catalytic Performance

The catalytic performance of MFM-300(Fe) was investigated in a fixed-bed reactor under flow conditions using a flow of 1–2% CH_4_ diluted in He as the feed gas at 25 °C, 1 atm. Compared with plasma alone and in the absence of catalyst, the catalytic performance was improved with Fe-based catalysts, including other Fe-MOFs, Fe-doped zeolite, and iron oxides (Table 1 and Figure S14). Overall, the Fe-MOFs and Fe/ZSM-5 show higher time yields than iron oxides, benefiting from their higher surface area (Figure S3).^24−26^ MFM-300(Fe) exhibits the highest time yield and C_2+_ products selectivity of 334 μmol g_cat_^–1^ h^–1^ and 96%, respectively, at a CH_4_ conversion of 3%. Only unsaturated C_2_ products (C_2_H_2_ and C_2_H_4_) were produced over MFM-300(Fe) at a low specific energy input of 2 kJ L^–1^, with no C_2_H_6_ detected under these conditions. The amount of H_2_ produced is consistent with that predicted based upon the productivity of C_2_H_2_ and C_2_H_4_ (Figure S15), representing an approach to H_2_ production from natural gas. Significantly, at a higher CH_4_ conversion of 10% at a specific energy input of 8 kJ L^–1^, the selectivity for total C_2+_ hydrocarbons (including C_2_ and C_3_) reached 98% with a superior time yield of 2056 μmol g_cat_^–1^ h^–1^ (Table S3). Thus, MFM-300(Fe) resolves the trade-off between CH_4_ conversion and product selectivity and represents to the best of our knowledge the first example of a MOF catalyst for non-oxidative coupling of CH_4_ to C_2+_ hydrocarbons. Control experiments (i) without CH_4_; (ii) without NTP at 25 °C; and (iii) without NTP but with heating at 250 °C, all yielded negligible amounts of C_2+_ products (Table 1). This confirms that NTP plays an important role in the activation of CH_4_ and that CH_4_ is the carbon source for C_2+_ products. The latter is confirmed further by isotopic labeling experiments using ^13^CH_4_/^12^CH_4_ as reactants (Figure S16).

**Table 1:** Summary of the NTP-Assisted Catalytic Performance for Conversion of CH_4_ over Different Catalysts^a^

		selectivity of products (%)					
entry	catalysts	C_2_H_2_	C_2_H_4_	C_2_H_6_	C_2+_ (C_2_ + C_3_)	unsaturated C_2_	time yield (μmol_C2+_ g_cat_^–1^ h^–1^)
1	empty tube	17 ± 1	47 ± 1	22 ± 1	90 ± 1	64 ± 1	
2	MFM-300(Fe)	38 ± 5	58 ± 6	0	96 ± 1	96 ± 1	334 ± 21
3	MFM-300(Fe) without CH_4_^b^	0	0	0	0	0	0
4	MFM-300(Fe)						
	NTP-off^c^	0	0	0	0	0	0
5	MFM-300(Fe)						
	NTP-off at 250 °C^d^	0	0	0	0	0	0
6	MIL-53(Fe)	18 ± 1	50 ± 1	25 ± 3	93 ± 1	68 ± 2	153 ± 25
7	MIL-100(Fe)	18 ± 2	48 ± 2	27 ± 3	94 ± 1	66 ± 4	197 ± 6
8	Fe/ZSM-5	18 ± 1	50 ± 1	23 ± 1	91 ± 1	68 ± 1	168 ± 12
9	Fe_2_O_3_	16 ± 1	47 ± 1	24 ± 1	91 ± 1	63 ± 1	138 ± 9
10	Fe_3_O_4_	15 ± 1	51 ± 1	24 ± 1	92 ± 1	66 ± 1	141 ± 9

a Reaction conditions: 1% CH_4_ diluted in He with a total flow rate of 60 mL min^–1^ as the feed gas, 60 mg of catalysts, 25 °C, and 1 atm.

b Without CH_4_: pure He is used as the feed gas.

c NTP-off at 25 °C: with plasma turned-off, at 25 °C.

d NTP-off at 250 °C: with plasma turned-off, heating at 250 °C.

MFM-300(Fe) exhibits excellent catalytic stability with a time-on-stream (ToS) of over 550 min. The reusability of the catalyst was confirmed by the retained activity and selectivity over three cycles (Figures 1 and S17). Little change was observed in the PXRD patterns, N_2_ adsorption isotherms, and the SEM and HAADF-STEM images for fresh and used catalysts (Figures S18–S20). XPS and XANES studies further confirmed retention of the oxidation state of the Fe(III) sites in the used catalyst (Figures S18 and S21), and EXAFS spectra confirmed (Figure S21 and Table S2) no change in the coordination environment at the Fe(III) centers. The Fe–O(H)–Fe sites are retained fully as confirmed by FTIR spectroscopic measurements (Figure S22).

### Fe–O(H)–Fe Sites for CH_4_ Binding and Activation

**Figure 2:**
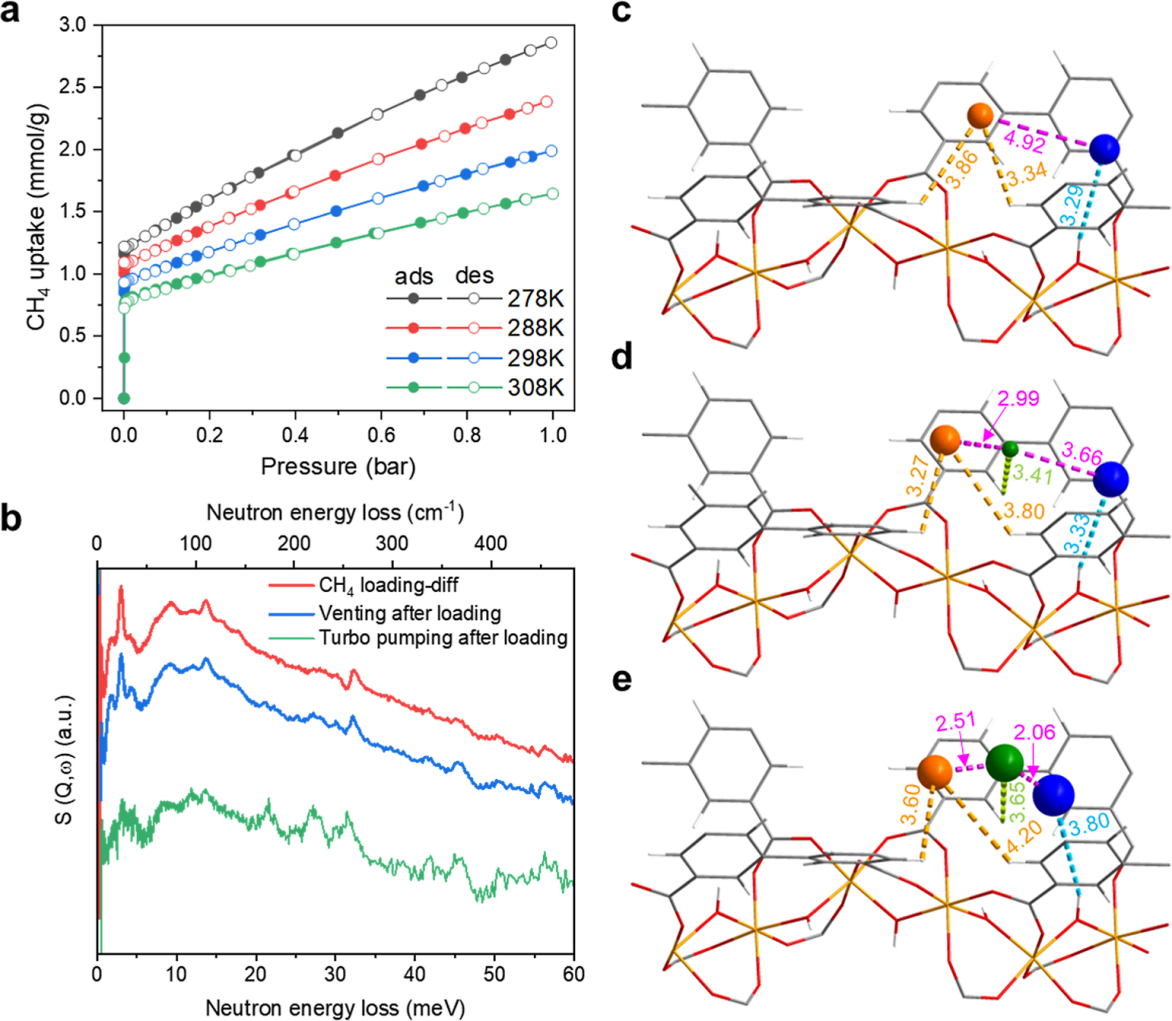
(a) Adsorption and desorption isotherms for CH_4_ in MFM-300(Fe). (b) Comparison of the difference INS spectra for CH_4_-loaded MFM-300(Fe) and that upon desorption under dynamic vacuum. Difference INS spectra were generated by subtraction of signals of the bare MOF and sample holder. S, dynamic structure factor; *Q*, momentum transfer; ω, frequency change. (c–e) Views of the binding sites for CD_4_ in (c) MFM-300(Fe)·(CD_4_)_1.9_, (d) MFM-300(Fe)·(CD_4_)_2.8_, and (e) MFM-300(Fe)·(CD_4_)_5.1_. All structures were derived from Rietveld refinement of NPD data collected at 7 K (C, gray; O, red; Fe, light orange; H, white; for CD_4_ molecules site I, blue; site II, orange; site III, green).

Adsorption isotherms of CH_4_ in MFM-300(Fe) at 278–308 K all show an initial steep rise and apparent incomplete desorption (residue of 0.23 CH_4_ per Fe) under pressure swing conditions (Figure 2a), indicating a binding affinity of MFM-300(Fe) for CH_4_. The binding domains of CD_4_ within MFM-300(Fe) have been determined by in situ NPD (Figures 2 and S23). At low loading (0.95 CD_4_ per Fe), two independent sites (CD_4_^I^ and CD_4_^II^) were observed. The primary binding site CD_4_^I^ (occupancy of 0.51 CD_4_ per Fe) is stabilized by the Fe–O(H)–Fe site, Fe–OH···CD_4_^I^ = 3.29(1) Å (Figures 2c and S23a). DFT calculations suggest that the system energy is reduced by 4.9 kJ mol^–1^ upon adsorption of CH_4_^I^ at the Fe–O(H)–Fe sites, confirming their unique role in lowering the energy barrier for C–H bond activation. CD_4_^II^ (occupancy of 0.44 CD_4_ per Fe) is located interstitially between two phenyl rings from ligands, C^II^···phenyl rings = 3.34(1) and 3.86(1) Å. The intermolecular distance between CD_4_^I^ and CD_4_^II^ is 4.92(1) Å. Upon further loading of CD_4_ (1.40 CD_4_ per Fe), the occupancies of CD_4_ molecules at both sites increase (Figures 2d and S23b), and the distance between CD_4_^I^ and the Fe–O(H)–Fe site also increases slightly, Fe–OH···CD_4_^I^ = 3.33(1) Å. Additional loading of CD_4_ results in a third binding site (CD_4_^III^), which is sandwiched by the CD_4_ molecules at sites I and II, CD_4_^I^···CD_4_^III^ = 3.66(2) Å and CD_4_^III^···CD_4_^II^ = 2.99(2) Å. At high loading (2.55 CD_4_ per Fe), the occupancies of CD_4_ molecules at all three sites increase, with a notable increase in the Fe–OH···CD_4_^I^ distance of 3.80(1) Å (Figures 2e and S23c); the distances CD_4_^I^···CD_4_^III^ and CD_4_^III^···CD_4_^II^ decrease to 2.06(1) and 2.51(1) Å, respectively. Interestingly, the overall distances Fe–OH···CD_4_^I^ in MFM-300(Fe) are shorter than those in MFM-300(In), which is isostructural to MFM-300(Fe) and a promising adsorbent for CH_4_,^27^ suggesting a stronger binding of CH_4_ to the Fe–O(H)–Fe sites.

INS studies, coupled with DFT calculations, enabled the direct visualization of the binding dynamics of CH_4_ in MFM-300(Fe) (Figures 2b and S24). Upon loading of CH_4_ (1.0 CH_4_ per Fe), the difference spectra show sharp features in the low-energy region (<40 meV), which are correlated to the translational and rotational motions of CH_4_ molecules (Figure 2b). The INS peaks at 55–60 meV are assigned to the change of O–H wagging modes, consistent with host–guest interaction between CH_4_ molecules and Fe–O(H)–Fe sites. Interestingly, these INS features are retained upon evacuating the CH_4_-loaded MFM-300(Fe) under dynamic vacuum at room temperature, confirming the binding of CH_4_ molecules to the framework. The integrated intensity of the entire INS spectra (including both elastic and inelastic scattering) confirms the retention of around 0.26 CH_4_ per Fe site, in excellent agreement with the data from the gas isotherm (0.23 CH_4_ per Fe). The host–guest interactions between CH_4_ molecules and the Fe–O(H)–Fe site in MFM-300(Fe) effectively lower the energy barrier for C–H bond activation and facilitate the catalytic CH_4_ conversion.

### Investigation of the Reaction Mechanism

**Figure 3:**
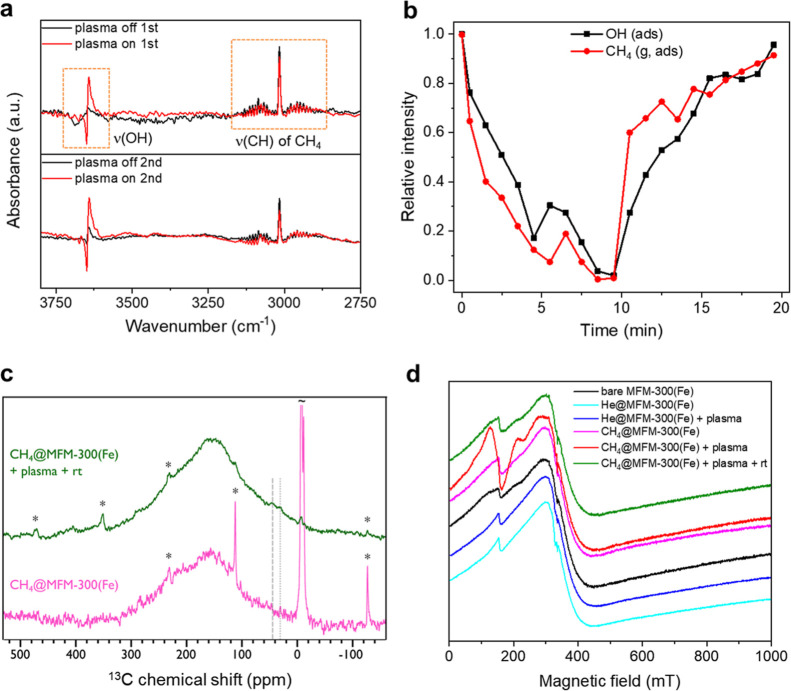
(a) In situ DRIFT spectra of MFM-300(Fe) in NTP-assisted CH_4_ conversion as a function of plasma off (black) and on (red); 1% CH_4_ diluted in He was used as the gas feed. All DRIFT spectra were recorded at a resolution of 4 cm^–1^, and the spectrum of bare MOF has been subtracted. (b) Correlation between the ν(OH) at 3649 cm^–1^ and ν(CH) at 3016 cm^–1^ as a function of plasma on–off and reaction time. Plasma was switched on at 0 min and switched off at 9.5 min. (c) ^13^C magic-angle spinning NMR spectra of CH_4_-loaded MFM-300(Fe) (bottom) and CH_4_-loaded MFM-300(Fe) after plasma treatment (top). Asterisks denote spinning sidebands (12 kHz separation) and vertical gray dashed and dotted lines highlight the presence of species at 45 and 31 ppm, respectively. The CH_4_ used for solid-state NMR experiments is ^13^C labeled. (d) In situ X-band (9.4 GHz) EPR spectra recorded at 20 K of the fresh and used MFM-300(Fe) catalyst after plasma treatment. The used catalyst was exposed to air at room temperature for 10 min.

Stabilization of reaction intermediates is a key step to suppress complete dehydrogenation of CH_4_ and over-oxidation to CO_2_. In situ DRIFT, solid-state NMR, and EPR spectroscopies afford valuable insights into the activation of CH_4_ molecules and the formation and stabilization of reaction intermediates. In situ plasma-DRIFTS experiments were carried out as a function of plasma off-on and reaction time under reaction conditions. Prior to the ignition of plasma, the differences in the DRIFT spectra (Figure 3a, top) show clear fundamental characteristic bands (C–H stretching)^28^ of CH_4_ at 3016 cm^–1^. The intensity of the IR band at 3016 cm^–1^ decreases upon ignition of plasma due to the activation and consumption of CH_4_. Meanwhile, a notable change is observed at ∼3649 cm^–1^, suggesting the red shift of the μ_2_-OH stretching mode in MFM-300(Fe) (Figures 3a and S22a). This indicates that the generated reaction intermediates are stabilized by the Fe–O(H)–Fe sites and converted to C_2+_ products, as confirmed by DRIFTS-MS studies (Figure S22). The close correlation between ν(CH) and ν(OH) bands confirms that CH_4_ molecules are activated and converted at the Fe–O(H)–Fe sites (Figures 3b and S25). In comparison, MIL-53(Fe) and Fe/ZSM-5 do not show such changes under the same conditions (Figure S26), consistent with their poor catalytic performance. Importantly, the changes in IR bands disappear upon switching plasma off and reappear upon switching plasma back on (Figure 3a), thus confirming the reversible nature of the catalytic process and the robustness and reusability of MFM-300(Fe).

The ^13^C ssNMR spectra for CH_4_-loaded MFM-300(Fe) before and after plasma treatment (Figures 3c and S28) provide further evidence to support the above findings. Peaks at δ{^13^C} = −6.9 and −11.1 ppm observed before plasma treatment indicate the presence of CH_4_ adsorbed at the acidic Fe–O(H)–Fe sites as well as gaseous species, respectively.^29^ After plasma treatment, these peaks are dramatically reduced, as would be expected for CH_4_ consumption, with only a small amount of adsorbed CH_4_ on these acidic sites remaining. It is clear that this remaining CH_4_ is in the proximity of paramagnetic Fe(III) centers owing to the extensive sidebands that span over 1000 ppm (Figure S28). Two new peaks also appear after plasma treatment, at δ{^13^C} = 45 and 31 ppm. The former can be assigned to a surface-bound methoxy group as an Fe–O(CH_3_)–Fe site.^30^ The latter is tentatively assigned to an ethylene oxide Fe–O(CH_2_CH_2_)–Fe species owing to the chemical shift, although the presence of 1,2-ethanediol Fe–OCH_2_CH_2_O–Fe species cannot be ruled out, which would demonstrate the importance of the *cis*-μ_2_-OH groups in MFM-300(Fe).

X-band EPR spectra for bare and CH_4_-loaded MFM-300(Fe) before and after plasma treatment all show a strong and very broad EPR signal centered at *g* ≈ 2, typical of paramagnetic Fe(III) centers (Figure 3d). The intensity of the broad signal decreases with reduction in temperature due to the antiferromagnetic exchange interaction between paramagnetic Fe(III) ions.^31^ All spectra demonstrate the presence of a very weak additional peak at *g* = 4.3 (Figures 3d and S29), likely due to distortions in the octahedral geometry of Fe(III) ions.^31^ Comparison of the EPR spectra for CH_4_-loaded MFM-300(Fe) before and after plasma treatment (Figure 3d) reveals an enhanced intensity of the peak at *g* = 4.3, indicating increased distortions around Fe(III) centers. These findings support the plasma-DRIFT and ssNMR spectroscopic results showing the formation and stabilization of methyl species on Fe–O(H)–Fe sites upon activation of the plasma. Interestingly, the increased intensity of the peak at *g* = 4.3 returned to its initial level when the sample was left at room temperature for 10 min, suggesting that these changes in EPR signals originate from short-lived intermediate states rather than decomposition of the MOF structure on plasma treatment. Overall, in situ plasma-DRIFT, NMR, and EPR studies confirm the formation of a series of reaction intermediates stabilized by the Fe–O(H)–Fe sites which are then converted to the desired C_2+_ products.

### Online Separation of C_2_H_4_ and C_2_H_2_ from Unreacted CH_4_

**Figure 4:**
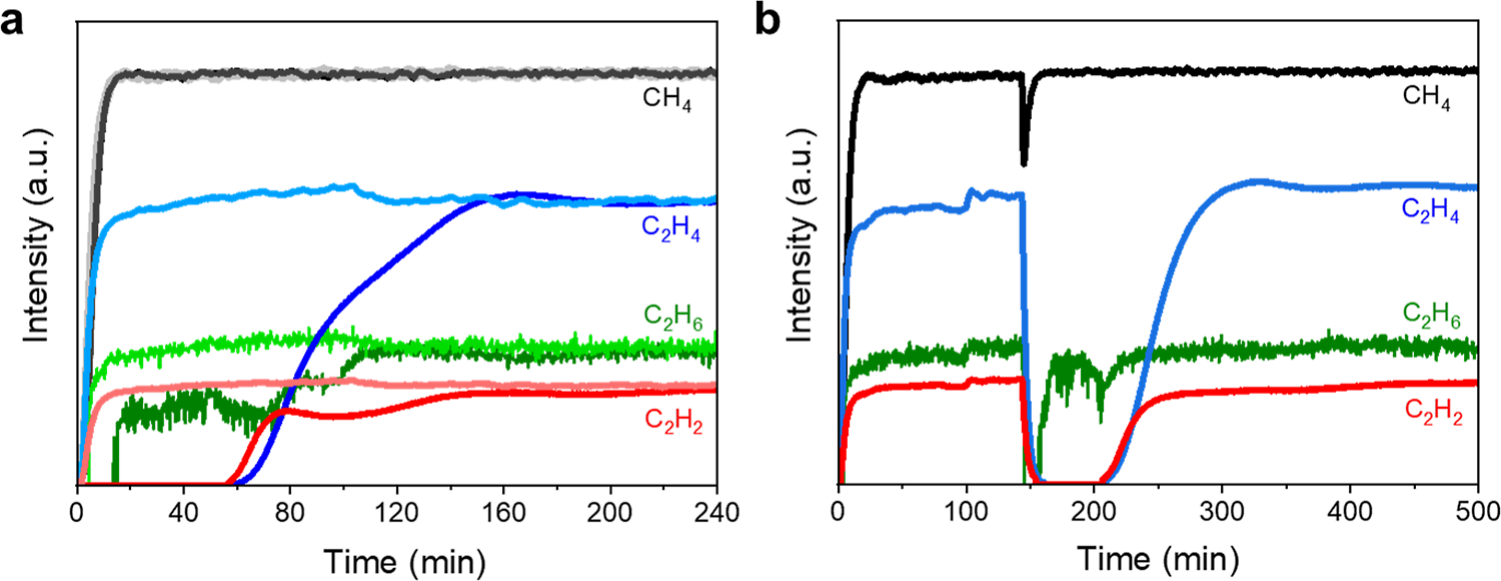
(a) Breakthrough curves showing the collection and the subsequent separation of generated C_2+_ products using HKUST-1. Light gray (CH_4_), light blue (C_2_H_4_), light green (C_2_H_6_), and light red (C_2_H_2_) curves were recorded at the exit of the reactor with no separation. Black (CH_4_), blue (C_2_H_4_), green (C_2_H_6_), and red (C_2_H_2_) curves were recorded after passing the exit stream through fixed bed-2 packed with HKUST-1 to separate C_2+_ products from CH_4_. (b) Switching exit stream from line 1 to line 2 (Figure S1) at 140 min during reaction. See Figures 1 and S1 for details of the experimental setup.

The separation of desired C_2+_ products from large amounts of unreacted CH_4_ is vitally important for practical applications since the conversion of CH_4_ in most state-of-the-art systems is typically <10%; this issue is, however, often overlooked. In our system, a cascade fixed-bed packed with benchmark sorbents (HKUST-1 or ZSM-5, Figures 4 and S30) has been developed to enable the online separation and collection of C_2_H_4_ and C_2_H_2_ from CH_4_ (Figures 1a and S1). Unreacted CH_4_ can pass readily through the sorbent-bed, feeding into a potential looping system for further delivery and conversion of CH_4_. Considering the rapidly increasing availability of porous materials with designed active sites, pore geometry, and ultrahigh selectivity to specific hydrocarbons, it is anticipated that the C_2+_ products can be collected with high purity by packing multiple, task-specific sorbents in the cascade fixed-beds.^32−34^

## Conclusions

In summary, we have established a promising system for the efficient activation and direct conversion of CH_4_ to C_2_H_4_ and C_2_H_2_ under ambient conditions and the online separation of desired products. We have overcome three major barriers in the activation and catalytic conversion of CH_4_: (i) creating highly active Fe–O(H)–Fe sites for direct activation of CH_4_ as well as stabilization of intermediates, greatly promoting the catalytic activity and selectivity; (ii) developing a cascade system for online separation of the multiple hydrocarbon products from unreacted CH_4_; and (iii) clarifying the critical roles of active Fe–O(H)–Fe sites within MFM-300(Fe) in the CH_4_ catalysis process via a series of complementary experimental and modeling studies. Combining the advantages of high CH_4_ conversion, high selectivity for C_2+_ unsaturated products, ambient operating conditions, ease of products separation from unreacted CH_4_, and detailed mechanistic studies, our system offers a potential practical solution to the future synthesis of chemicals and materials from abundant natural gas.

## Experimental Section

### EPR Spectroscopy

EPR spectroscopy was performed at 100–10 K on a Bruker EMX 300 EPR spectrometer equipped with an X-band (ca. 9.4 GHz) resonator and a liquid He cryostat. Fine powder (10–15 mg) of MFM-300(Fe) was placed in 4 mm quartz tubes fitted with a J. Young tap. To generate the desolvated samples, the J. Young tube was connected to a high vacuum line (10^–5^ mbar) for 2 h at room tempertature, followed by 15 h at 150 °C. The desolvated samples in the J. Young tubes were dosed with CH_4_ and/or He gas. After gas dosing, the sample tubes were sealed for EPR measurements. The gas-loaded samples were further treated with plasma and preserved in liquid N_2_ before EPR measurements. Spectra were recorded with a microwave power of 0.7–7 mW, modulation frequency of 100 kHz, and modulation amplitude of 10 G. Background spectra were collected on empty tubes and subtracted from all reported spectra, and field corrections applied by measuring relevant EPR standards (Bruker Strong Pitch and DPPH). Simulation of the EPR spectra was undertaken using the EasySpin package.^35^

### ssNMR Spectroscopy

Magic angle spinning (MAS) NMR spectroscopy was performed at ambient temperature on a Bruker 9.4 T (400 MHz ^1^H Larmor frequency) AVANCE III spectrometer equipped with a 4 mm HFX MAS probe. Desolvated samples were packed into 4 mm zirconia rotors and were then treated before being sealed with a Kel-F rotor cap. The treatment included ^13^CH_4_ loading, a short plasma treatment (∼1 min), and a long plasma treatment (∼5 min). Experiments were acquired using a MAS frequency of 12 kHz. ^1^H- and ^13^C-pulses of 100 and 50 kHz were used, respectively, for ^1^H and ^13^C direct excitation (DE)MAS experiments. A Hahn-echo τ_r_–π–τ_r_ sequence of 2 rotor periods total duration was applied for ^1^H before acquisition. Recycle delays of 1 and 5 s were used for ^1^H and ^13^C DEMAS experiments, respectively. ^1^H and ^13^C chemical shifts are given with respect to TMS (0 ppm).

### Neutron Powder Diffraction

NPD experiments were carried out at WISH, a long wavelength powder and single-crystal neutron diffractometer at the ISIS Facility at the Rutherford Appleton Laboratory, UK.^36^ Prior to NPD experiments, the sample was activated by heating at 473 K under dynamic vacuum, and the desolvated sample was then transferred into a cylindrical vanadium sample cell with an indium seal. The sample was further degassed at 373 K under dynamic vacuum to remove traces of adsorbed moisture. The temperature during data collection was controlled using a liquid He cryostat (7 ± 0.2 K). The quality of the Rietveld refinements was assured with low goodness-of-fit factors, low weighted profile factors (*R*_wp_), and well-fitted patterns with reasonable isotropic displacement factors.

### Inelastic Neutron Scattering

INS experiments were performed at the VISION spectrometer at Spallation Neutron Source, Oak Ridge National Laboratory, US. VISION is an indirect geometry crystal analyzer instrument that provides a wide dynamic range with high resolution. The desolvated MOF sample (1.45 g), which was prepared by heating the fresh sample at 473 K under dynamic vacuum, was loaded into a cylindrical vanadium sample container with an indium vacuum seal and connected to a gas handling system. The sample was further degassed at 393 K for 16 h to remove any remaining guest water molecules. The temperature during data collection was controlled by using a closed-cycle refrigerator cryostat (5 ± 0.1 K). The loading of CH_4_ was performed volumetrically at room temperature in order to ensure that CH_4_ was present in the gas phase when not adsorbed and also to ensure sufficient mobility of the gas inside the crystalline structure of the MOF. Subsequently, the temperature was reduced to 5 K in order to perform the scattering measurements with the minimum achievable thermal motion for the framework host and adsorbed CH_4_ molecules. Background spectrum (sample can plus bare MOF) was subtracted to obtain the difference spectra. INS spectrum for condensed CH_4_ in the solid state was measured by using a plate sample container. Approximately 1–2 L of CH_4_ at room temperature was condensed slowly at the temperature below its melting point and cooled to temperature below 7 K for neutron scattering measurements.

### Catalytic Performance Testing

The catalytic performance, including the conversion of CH_4_ and selectivity of C_2+_ products over the catalysts, were tested under NTP activation. A mixture of CH_4_ and He (1 and 2% CH_4_ diluted in He, 25 °C, 1 atm) at a total flow rate of 60 mL min^–1^ controlled by mass flow controllers was used as the feed gas. Prior to the NTP-assisted catalytic reaction, the catalysts were activated by heating at 150 °C under dynamic vacuum for 16 h. The desolvated sample (60 mg) was packed in a fixed-bed reactor^26,37^ and treated at 80 °C for 1 h under a flow of He (60 mL min^–1^) to remove any residual water in the system. A gas mixture of CH_4_ and He was then allowed to pass through the fixed-bed reactor to test the catalytic performance of each catalyst.

### Adsorption Isotherms for CH_4_

Gravimetric sorption isotherms for CH_4_ were recorded on a Hiden Isochema Intelligent Gravimetric Analyzer (IGA) system under ultra-high vacuum using a turbo pumping system. The temperature was maintained between 278 and 308 K using a temperature-programmed water bath. In a typical gas adsorption experiment, 50 mg of acetone-exchanged MOF was loaded into the IGA system and activated at 473 K under dynamic high vacuum (10^–8^ bar) for 24 h to give a fully desolvated sample.

## Supporting Information Available

Additional experimental details, PXRD, N_2_ isotherms, SEM, HAADF-STEM, views of crystal structures, and NPD, INS, DRIFTS, MS, XAFS, XPS, EPR, and ssNMR data (PDF)
