# Supplementary material for: Direct Conversion of Methane to Ethylene and Acetylene over an Iron-Based Metal–Organic Framework
Source: J Am Chem Soc. 2023 Sep 18;145(38):20792–800. doi: 10.1021/jacs.3c03935 (PMC10540182; doi:10.1021/jacs.3c03935)
Supplement: Supplementary file 1 — ja3c03935_si_001.pdf [file ja3c03935_si_001.pdf]

# Supporting Information

## Direct Conversion of Methane to Ethylene and Acetylene over an Iron-Based Metal-Organic Framework

Yujie Ma[1], Xue Han[1], Shaojun Xu[2], Zhe Li[3,4], Wanpeng Lu[1], Bing An[1], Daniel Lee[2], Sarayute Chansai[2], Alena M. Sheveleva[1,5], Zi Wang[1], Yinlin Chen[1], Jiangnan Li[1], Weiyao Li[1], Rongsheng Cai[6], Ivan da Silva[7], Yongqiang Cheng[8], Luke L. Daemen[8], Floriana Tuna[1,5], Eric J. L. McInnes[1,5], Lewis Hughes[9], Pascal Manuel[7], Anibal J. Ramirez-Cuesta[8], Sarah J. Haigh[6], Christopher Hardacre[2], Martin Schröder[1]* and Sihai Yang[1,10]*

[1]Department of Chemistry, University of Manchester, Manchester, M13 9PL, UK.

[2]Department of Chemical Engineering, University of Manchester, Manchester, M13 9PL, UK.

[3]The Francis Crick Institute, London, NW1 1AT, UK.

[4]Department of Chemistry, King's College London, London, WC2R 2LS, UK.

[5]Photon Science Institute, University of Manchester, Manchester, M13 9PL, UK.

[6]Department of Materials, University of Manchester, Manchester, M13 9PL, UK.

[7]ISIS Facility, Science and Technology Facilities Council, Rutherford Appleton Laboratory, Chilton, OX11 0QX, UK.

[8]Neutron Scattering Division, Neutron Sciences Directorate, Oak Ridge National Laboratory, Oak Ridge, Tennessee, 37831, USA.

[9]Department of Earth and Environmental Sciences, University of Manchester, Manchester, M13 9PL, UK.

[10]College of Chemistry and Molecular Engineering, Beijing National Laboratory for Molecular Sciences, Peking University, Beijing 100871, China

## Materials and Methods

### Preparation of the materials

All the reagents were used as received from commercial suppliers without further purification. $Fe_2O_3$, $Fe_3O_4$ and ZSM-5 were purchased from Alfa Aesar.

**Synthesis of MFM-300(Fe).** MFM-300(Fe) was synthesised using our previously reported method.[1] Typically, 244 mg of biphenyl-3,3′,5,5′-tetracarboxylic acid and 800 mg of $FeCl_3 \cdot 6H_2O$ were dispersed in a mixture solution of N,N-dimethylformamide (DMF, 20 mL) and concentrated hydrochloric acid (0.75 mL) in a 50 mL round-bottom flask under ambient pressure. The reaction was heated at 120 °C for 72 h, and the yellow powder product was collected and washed with DMF and acetone several times. The as-synthesised MFM-300(Fe) was dried and activated by heating at 150 °C under dynamic vacuum before further use.

**Synthesis of MIL-53(Fe).** MIL-53(Fe) was synthesised by a solvothermal method according to the reported method.[2] Typically, 0.66 g of terephthalic acid ($H_2BDC$) and 1.08 g of iron(III) chloride hexahydrate ($FeCl_3 \cdot 6H_2O$) were dissolved in 20 mL of DMF. The mixture was transferred into a teflon lined bomb, sealed and heated at 150 °C for 24 h. The product was collected by centrifugation and washed with DMF and deionized water several times. The as-synthesised MIL-53(Fe) was dried and activated by heating at 150 °C under dynamic vacuum before further use.

**Synthesis of MIL-100(Fe).** MIL-100(Fe) was synthesised by a solvothermal method according to the reported method.[3] Typically, 0.32 g of trimesic acid ($H_3BTC$) and 0.68 g of iron(III) nitrate nonahydrate [$Fe(NO_3)_3 \cdot 9H_2O$] were dissolved in a mixture solution containing 10 mL of deionized water and 0.18 mL of hydrofluoric acid. The mixture was transferred into a teflon lined bomb, sealed and heated at 150 °C for 12 h. The product was collected by centrifugation and washed with deionized water and EtOH several times. The as-synthesised MIL-100(Fe) was dried and activated by heating at 150 °C under dynamic vacuum before further use.

**Synthesis of Fe/ZSM-5.** Fe/ZSM-5 was synthesised according to a reported method.[4] Typically, 500 mg of ZSM-5 was added into a solution containing $Fe(NO_3)_3 \cdot 9H_2O$ (800 mg) dissolved in deionized water (20 mL), and the mixture was then heated at 85 °C for 24 h. The product was centrifuged, washed with deionized water several times, dried overnight at 80 °C and finally calcined at 550 °C under air flow for 3h.

**Synthesis of HKUST-1.** HKUST-1 was synthesised through solvothermal method according to the reported method.[5,6] Typically, 0.84 g of $H_3BTC$ and 1.45 g of copper(II) nitrate trihydrate

$[Cu(NO_3)_2{\cdot}(H_2O)_3]$ were dissolved in a mixture solution containing DMF (20 mL), EtOH (20 mL) and deionized water (20 mL). The mixture was transferred into a round bottom flask, then heated at 85 °C for 12 h. The product was collected by centrifugation and washed with DMF and EtOH several times. The as-synthesised HKUST-1 was dried and activated by heating at 150 °C under dynamic vacuum before further use.

**General characterisation of the materials**

Powder X-ray diffraction (PXRD) patterns were recorded on a Philips X'pert X-ray diffractometer (40kV and 30 mA) using Cu-Kα radiation (λ = 1.5406 Å). Nitrogen adsorption isotherms were collected on a Micromeritics 3Flex analyzer at 77 K. The samples were activated under dynamic vacuum before measuring $N_2$ isotherms. The specific surface area was calculated using the Brunauer-Emmett-Teller (BET) method. Scanning electron microscopy (SEM) imaging and energy dispersive X-ray spectroscopy (EDX) analysis was performed using the FEI/Thermofisher Quanta 650 field emission gun SEM at the University of Manchester. The SEM was equipped with a Bruker X Flash 6 | 30 silicon drift detector with Bruker ESPRIT EDX software v2.2. For high-resolution imaging, beam deceleration was employed to achieve a landing energy of 1 kV. For EDX analysis, beam conditions were set to 15 kV. High-angle annular dark-field scanning transmission electron microscopy (HAADF-STEM) images and EDX elemental maps were collected on a Thermo Fisher Titan STEM (G2 80-200) equipped with a Cs probe corrector (CEOS), high-angle annual dark-field (HAADF) detector and ChemiSTEM Super-X EDX detector, operating at 200 kV. TEM samples were prepared by dispersing the powders in methanol and drop-cast onto a copper grid coated with an amorphous holey carbon film. X-ray photoelectron spectroscopy (XPS) was performed using an Axis Ultra Hybrid spectrometer (Kratos Analytical, Manchester, United Kingdom) using monochromated Al-Kα radiation (1486.6 eV, 10 mA emission at 150 W, spot size 300 x 700 μm) with a base vacuum pressure of $\sim 5\times10^{-9}$ mbar. Charge neutralisation was achieved using a filament. Binding energy scale calibration was performed using C-C in the C 1s photoelectron peak at 284.8 eV. Analysis and curve fitting was performed using Voigt-approximation peaks using CasaXPS.

**Inelastic neutron scattering (INS)**

Direct visualisation of the interactions between adsorbed $CH_4$ molecules and the active sites in MFM-300(Fe) is crucial to understanding the molecular details of adsorption, activation and conversion of $CH_4$ over catalyst. INS is a powerful neutron spectroscopy technique[7] to investigate the dynamics (particularly for H-containing compounds, such as hydrocarbons) of host-guest interactions because it has several unique advantages:

- INS spectroscopy is ultra-sensitive to the vibrations of hydrogen atoms, and hydrogen is ten times more visible than other elements due to its high neutron cross-section.
- The technique is not subject to any optical selection rules. All vibrations are active and, in principle, measurable.
- INS observations are not restricted to the centre of the Brillouin zone (gamma point) as is the case for optical techniques.
- INS spectra can be readily and accurately modelled: the intensities are proportional to the concentration of elements in the sample and their cross-sections, and the measured INS intensities relate straightforwardly to the associated displacements of the scattering atom. Treatment of background correction is also straightforward.
- Neutrons penetrate deeply into materials and pass readily through the walls of metal containers making neutrons ideal to measure bulk properties of this material.
- INS spectrometers cover the whole range of the molecular vibrational spectrum, 0-500 meV (0-4000 $cm^{-1}$).
- INS data can be collected at low temperature (below 10 K), where the thermal motion of the MOF material and the adsorbed $CH_4$ molecules is significantly reduced.
- Calculation of the INS spectra by DFT vibrational analysis can be readily achieved, and DFT calculations relate directly to the INS spectra, and, in the case of solid-state calculations, there are no approximations other than the use of DFT eigenvectors and eigenvalues to determine the spectral intensities.

**Experimental setup for catalytic testing**

For the NTP-assisted reaction, the reactor comprised of two coaxial quartz tubes; the outside diameters of the outer tube and inner tube was 6 mm and 3 mm, respectively, giving a discharge gap of 0.5 mm.[8,9] The outer tube was covered by a metal mesh electrode that was connected to a

high-voltage output, and a metal wire electrode (ground electrode) was placed inside the inner tube. The catalyst was packed in the discharge region to ensure that plasma was generated around the catalyst. An alternating current plasma generator (CTP-2000K, 0-25 kV, 10 kHz) was used to ignite the plasma, and an oscilloscope (Tektronix TDS 2022B) was used to monitor the electrical parameters. The discharge power used for reaction was about 2 W and the specific energy input was calculated to be around 2 kJ $L^{-1}$, with the AC peak-to-peak voltage (Vpk-pk) at around 20 kV and a frequency of 10 kHz. At a high $CH_4$ conversion of ~10%, the specific energy input was calculated to be around 8 kJ $L^{-1}$.

The gaseous products were detected using (i) a Bruker MATRIX-MG5 FTIR spectrometer (resolution = 0.5 $cm^{-1}$) for $CH_4$, $C_{2+}$ products, CO and $CO_2$ and (ii) a mass spectrometer (Hiden QGA quantitative gas analysis system, Hiden Analytical Ltd.) for $H_2$. The Bruker MATRIX-MG5 features a 5 m multi-reflection gas cell and is designed for the high-precision quantification of gas compounds from very low concentrations on the ppb level up to one hundred percent. The gas analysis system uses the certain sections of FTIR spectrum that are unique to a given gas to first identify the gas is present and then uses a fitting algorithm to quantity the amount of gas present. If other gases interfere with the signal, then the system also fits these interfering gases and takes them into account when performing the quantification. The system was calibrated and set up before use.

### ***In situ*** **diffuse reflectance infrared Fourier transform spectroscopy (DRIFTS)**

To investigate the surface chemistry of the NTP-assisted $CH_4$ conversion reaction, *in situ* DRIFTS measurements were performed using a Bruker Vertex 70 FTIR spectrometer equipped with a liquid $N_2$-cooled detector, and a custom-made fixed-bed NTP-DRIFTS reactor.[10] Approximately 25 mg of MFM-300(Fe) sample was placed in a crucible in an *in situ* DRIFTS cell. The gas mixture was controlled by independent mass flow controllers and introduced to an *in situ* DRIFTS cell *via* a 4-way valve that allows a switch between He pre-treatment and $CH_4$ gas feed.

Prior to DRIFTS measurement, MFM-300(Fe) was pre-treated to remove any adsorbed water by heating up to 120 °C in He flow with a total flow rate of 50 mL $min^{-1}$. After 1 h, the temperature was decreased to room temperature and the IR spectrum of MFM-300(Fe) recorded as a background. Subsequently, the gas feed containing 1% $CH_4$ (diluted in He balance) was introduced to the NTP-DRIFTS reactor for 5 min before switching on the plasma. The power electrode was

driven at ~ 27 kHz with an applied peak voltage of 5.0 kV. The discharge electrical parameters were measured using a resistor (10 Ω) for the current and a high-impedance probe (Tektronix, P6015) for the applied voltage, connected to a digital oscilloscope (Tektronix TBS1072B). The cycling switches between NTP on and off were carried out every 10 min to monitor the effect of plasma on the changes of surface species. All DRIFT spectra were recorded with a resolution of 4 $cm^{-1}$ and an accumulation of 128 scans every 60 s and analysed by the OPUS software.

**Experimental setup for $C_{2+}$ products separation**

A cascade system was designed for the separation of the $C_{2+}$ hydrocarbon products from unreacted $CH_4$. Typically, the feed gas ($CH_4$ diluted in He) was passed through the fixed bed plasma reactor (fixed bed-1, see Figure S1 for details of the experimental setup), and $CH_4$ was partially converted to $C_{2+}$ products. When the gas mixture passes through line 1 (without the fixed bed-2 for $C_{2+}$ collection), the gas mixture is analysed using a Bruker Matrix MG5 FTIR spectrometer. When the gas mixture passes through line 2 where the fixed bed-2 is packed with porous materials (HKUST-1 or ZSM-5), $C_{2+}$ products can be separated and collected and the unreacted $CH_4$ detected by FTIR spectroscopy.

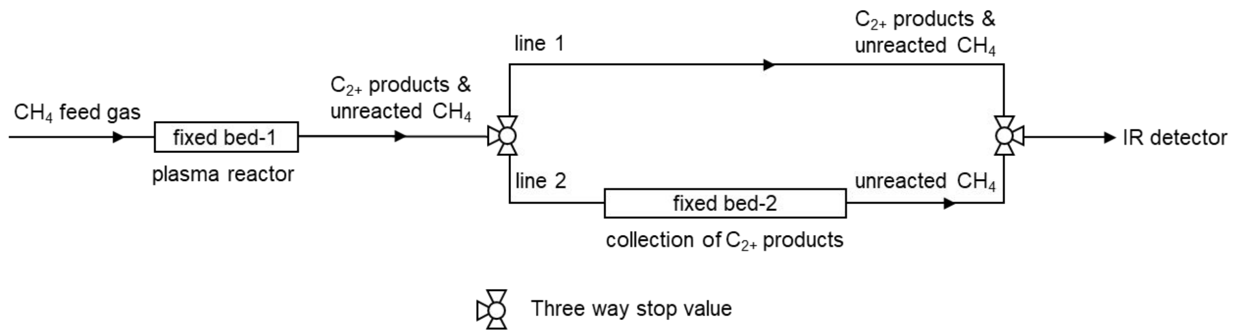


**Figure S1.** View of the experimental setup for the cascade system. Fixed bed-1 is packed with catalyst for conversion of $CH_4$ to $C_{2+}$ products, and fixed bed-2 is packed with porous materials (HKUST-1 or ZSM-5) for separation of $C_{2+}$ products from unreacted $CH_4$.

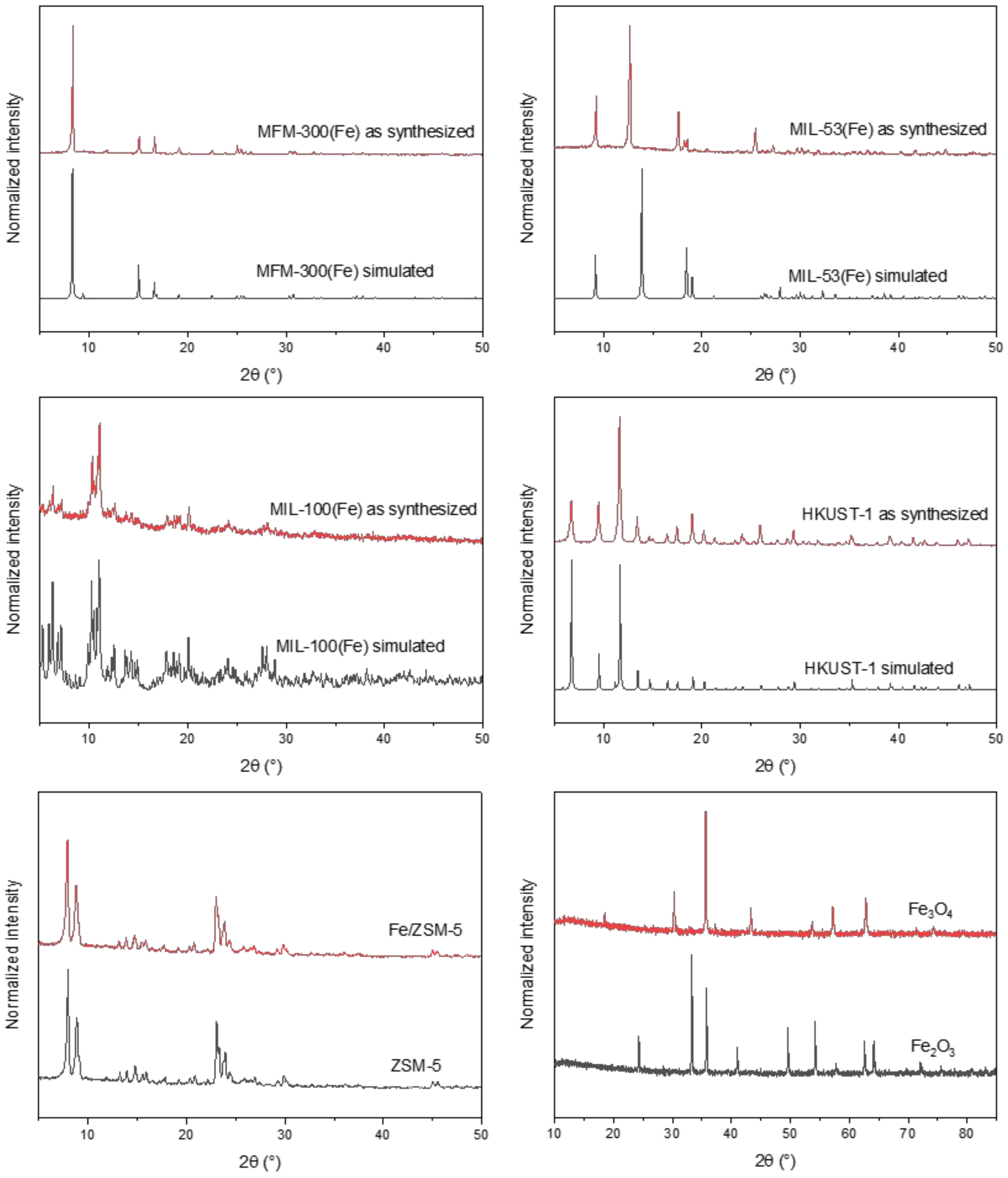


**Figure S2.** PXRD patterns of materials investigated.

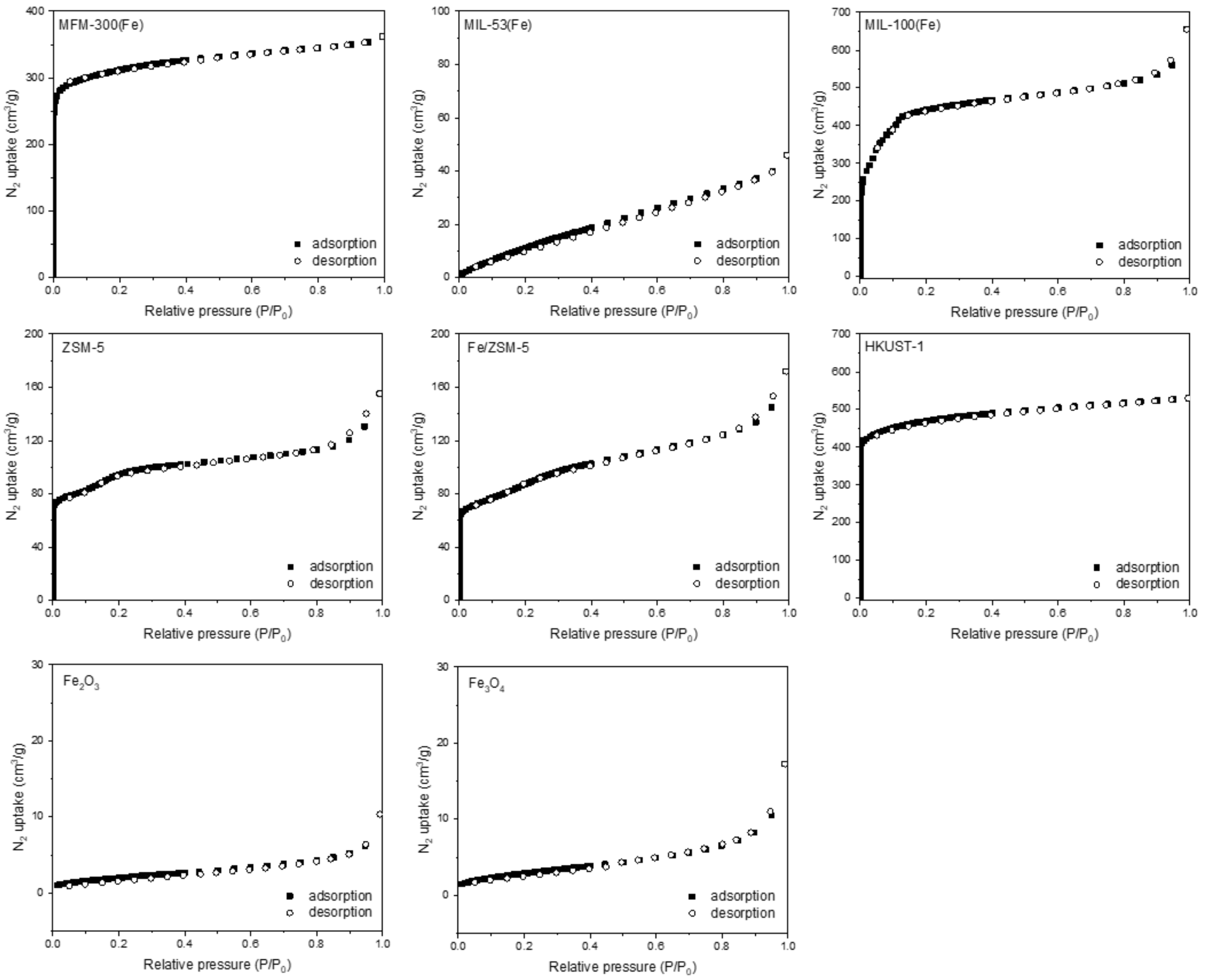


**Figure S3.** $N_2$ adsorption and desorption isotherms.

**Table S1.** Summary of BET surface areas determined from $N_2$ sorption isotherms.

| Sample | Calculated BET surface area ($m^2$ $g^{-1}$) |
|---|---|
| MFM-300(Fe) | 1056 |
| MIL-53(Fe) | 64 |
| MIL-100(Fe) | 1424 |
| ZSM-5 | 310 |
| Fe/ZSM-5 | 300 |
| HKUST-1 | 1468 |
| $Fe_2O_3$ | 8 |
| $Fe_3O_4$ | 11 |

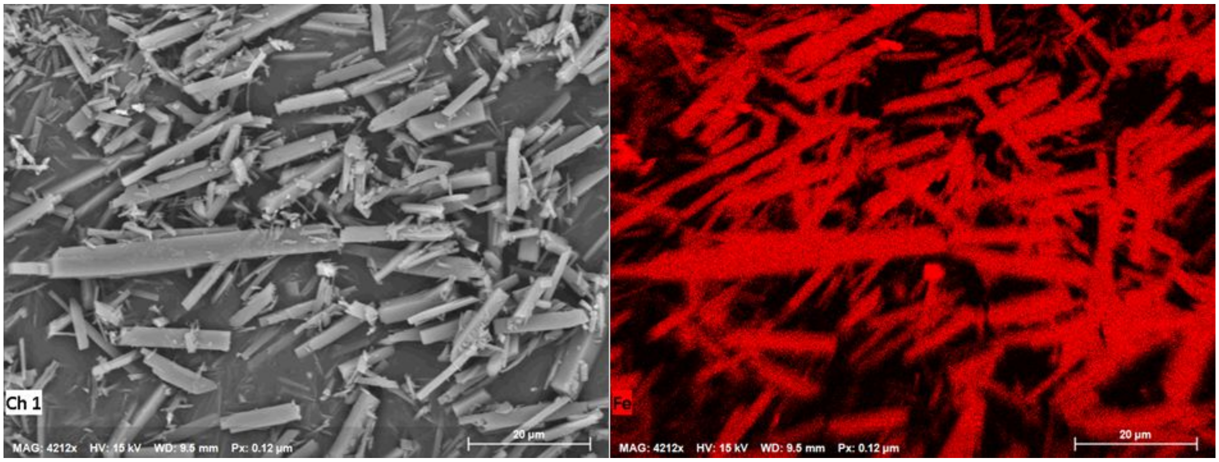


**Figure S4.** SEM image and EDX mapping of as-synthesised MFM-300(Fe).

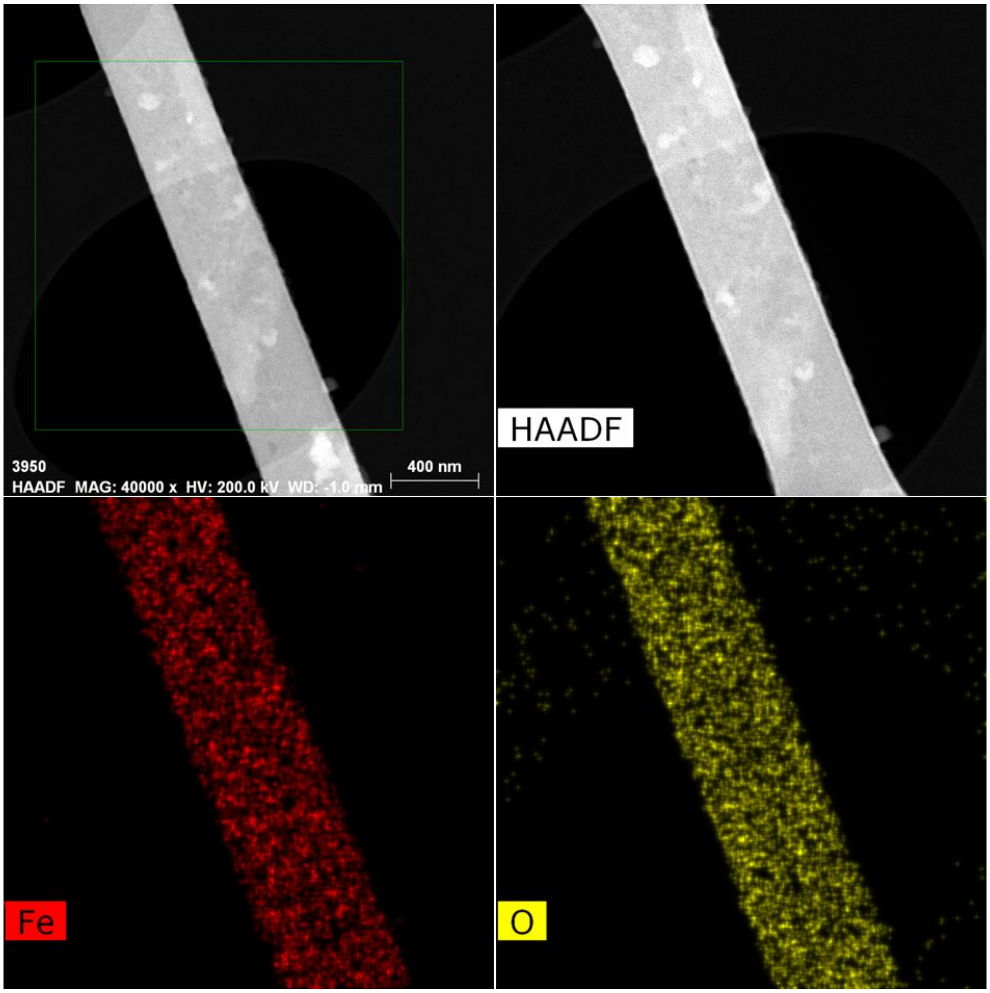


**Figure S5.** HAADF-STEM images and EDX mapping of as-synthesised MFM-300(Fe).

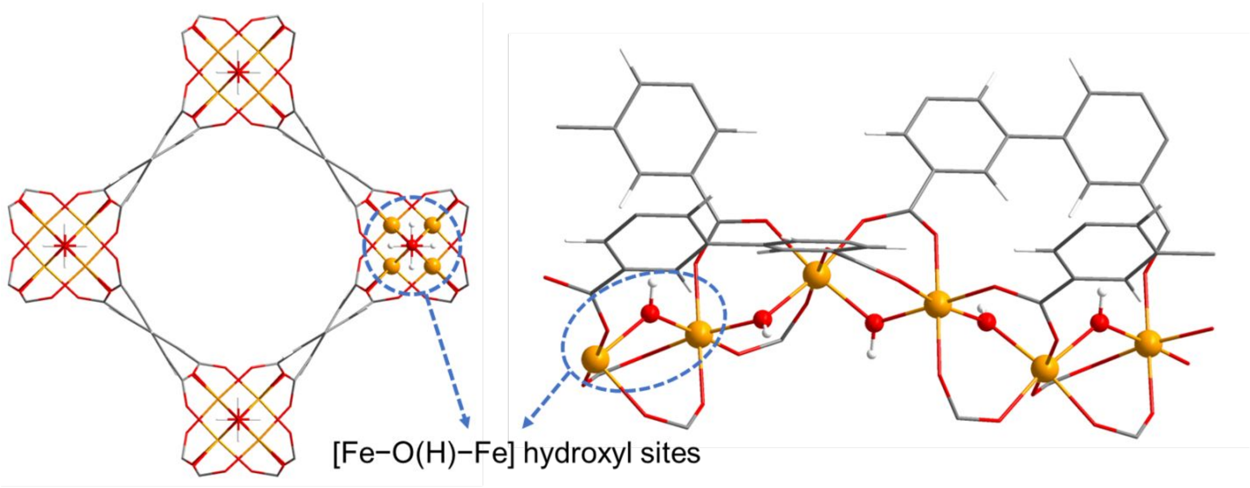


**Figure S6.** Views of the structure of MFM-300(Fe) determined by NPD at 7 K (C, grey; O, red; Fe, light orange; H, white). The Fe−O(H)−Fe hydroxyl sites are partially highlighted in ball-and stick model.

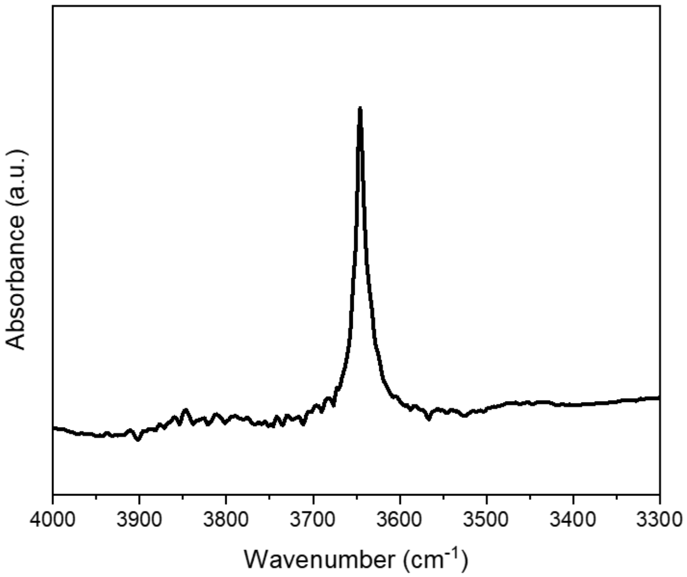


**Figure S7.** FTIR spectrum of MFM-300(Fe) in the region 4000-3300 $cm^{-1}$ showing the υ(O−H) stretching vibration.

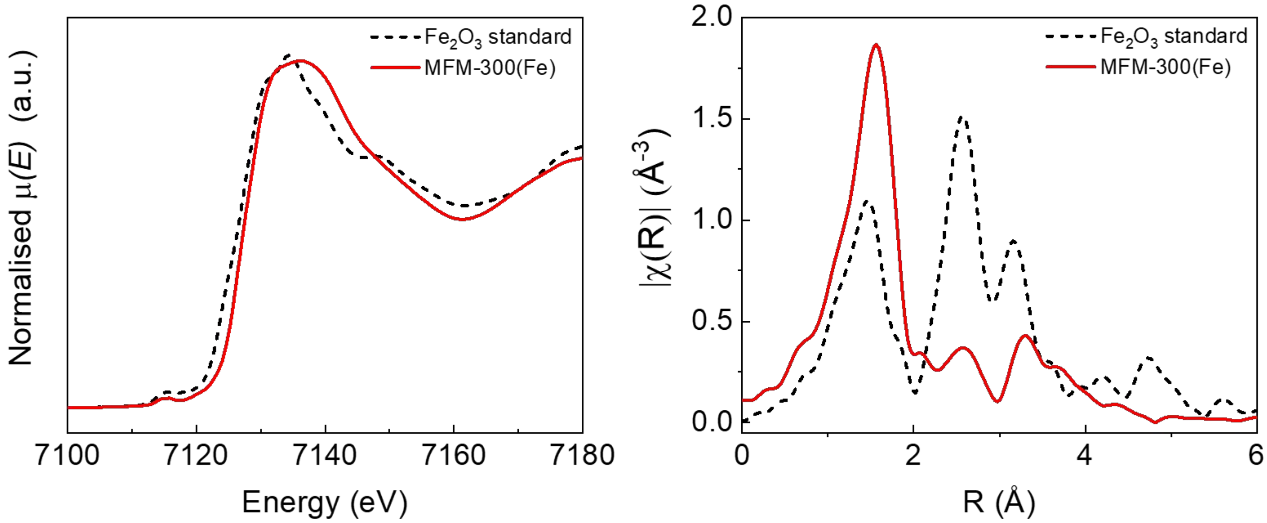


**Figure S8.** Normalised Fe *K*-edge XANES spectrum of as-synthesised MFM-300(Fe) and standard XANES spectrum of $Fe_2O_3$ reference (left). Plot of non-phase corrected Fourier transformed Fe *K*-edge EXAFS data for as-synthesised MFM-300(Fe) shown against a $Fe_2O_3$ standard reference (right).

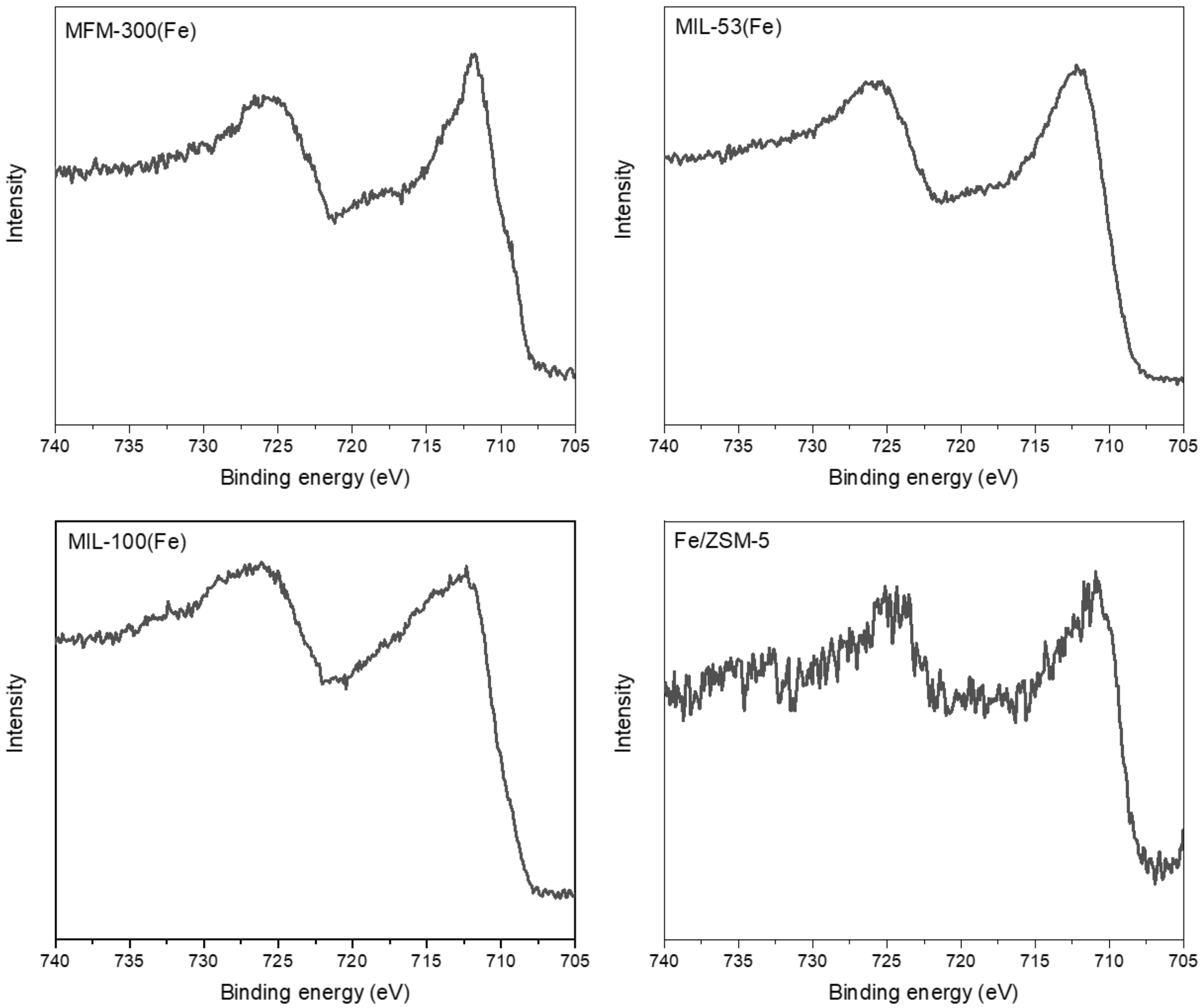


**Figure S9.** Fe 2p XPS spectra of the as-synthesised materials.

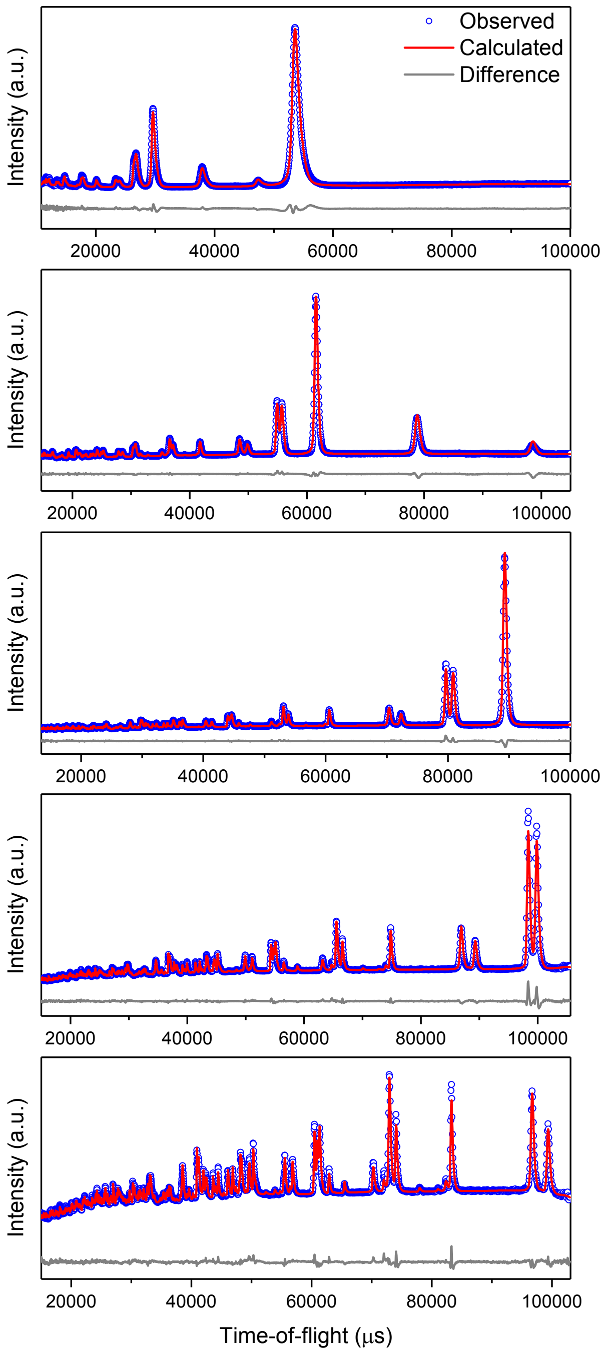


**Figure S10.** Neutron powder diffraction patterns and Rietveld refinement for bare MFM-300(Fe) (banks 1 to 5). Fitting agreement parameters: $R_{exp}$=0.44%; $R_{wp}$=1.66%; $R_p$=1.35%; Gof=3.77.

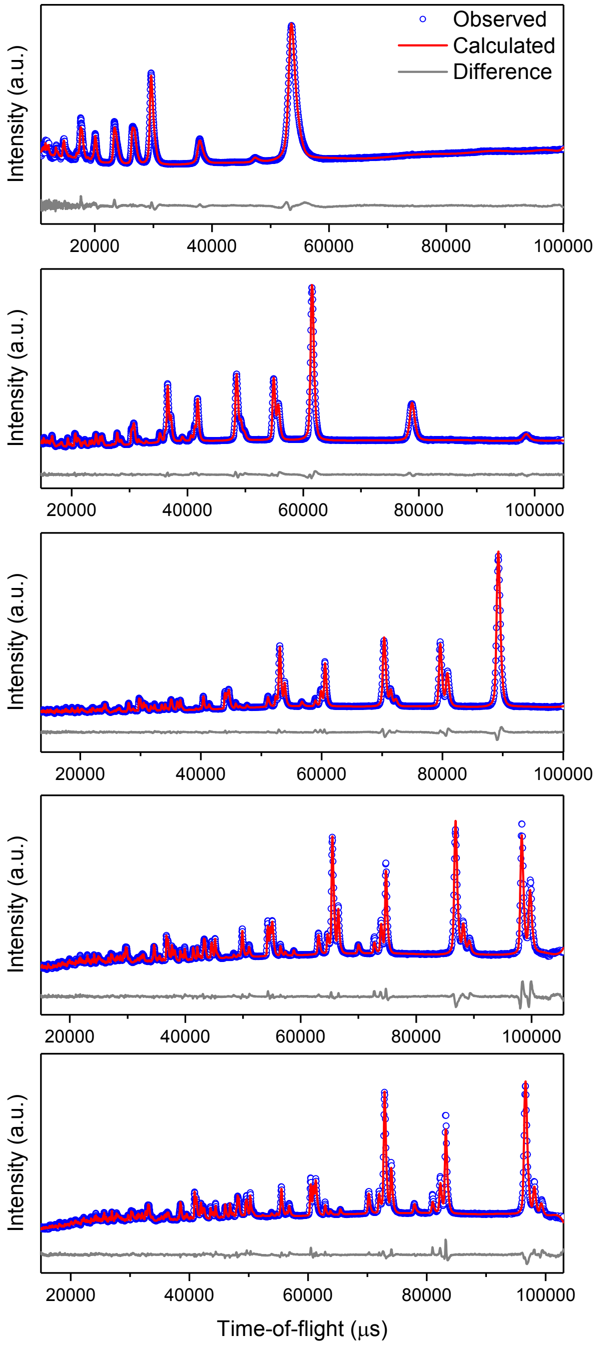


**Figure S11.** Neutron powder diffraction patterns and Rietveld refinement for MFM-300(Fe)·$(CD_4)_{1.9}$ (banks 1 to 5). Fitting agreement parameters: $R_{exp}$=0.43%; $R_{wp}$=1.68%; $R_p$=1.43%; Gof=3.92.

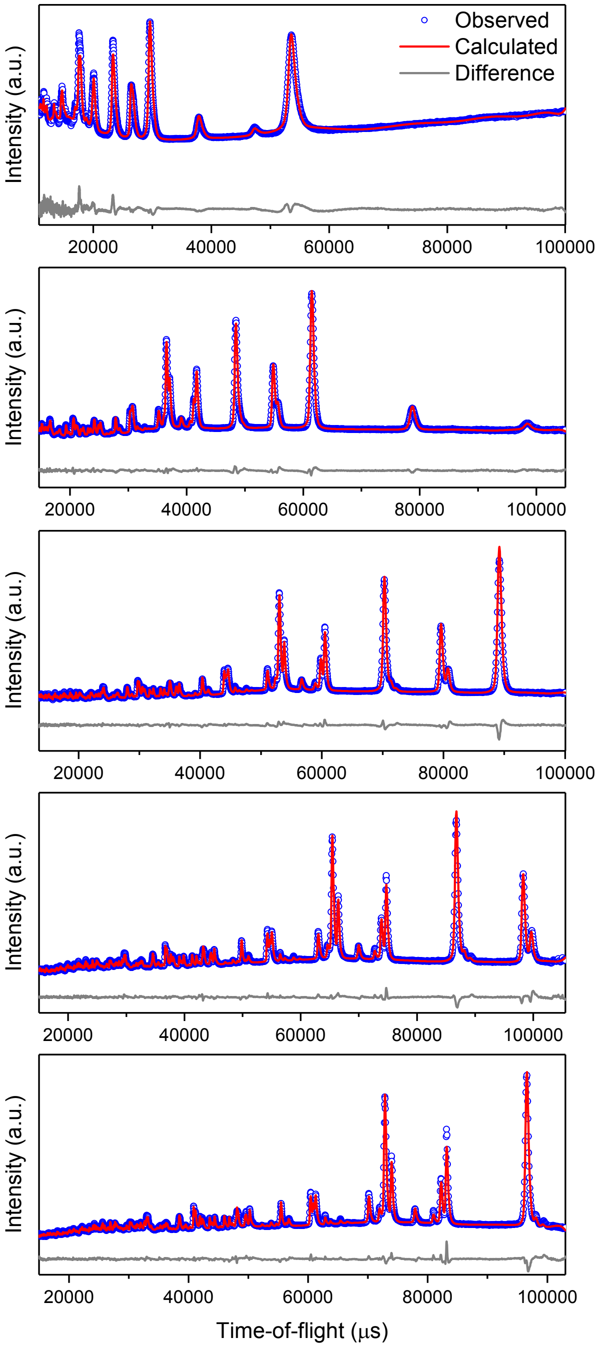


**Figure S12.** Neutron powder diffraction patterns and Rietveld refinement for MFM-300(Fe)·$(CD_4)_{2.8}$ (banks 1 to 5). Fitting agreement parameters: $R_{exp}$=0.42%; $R_{wp}$=1.90%; $R_p$=1.61%; Gof=4.50.

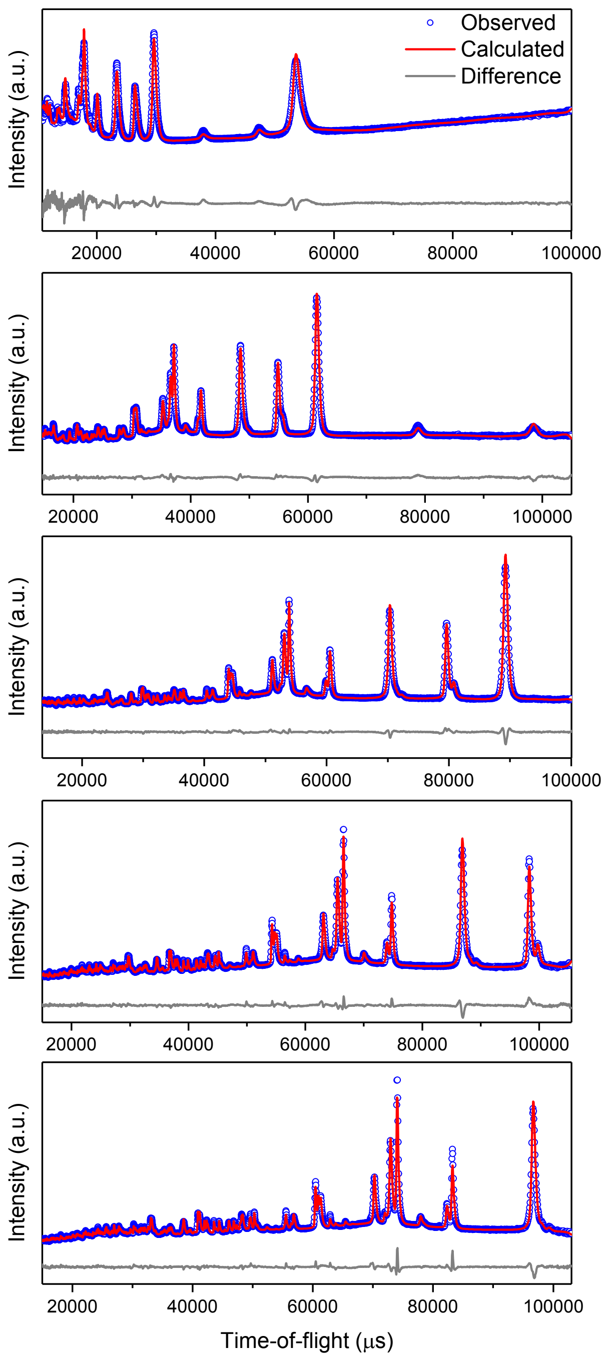


**Figure S13.** Neutron powder diffraction patterns and Rietveld refinement for MFM-300(Fe)·$(CD_4)_{5.1}$ (banks 1 to 5). Fitting agreement parameters: $R_{exp}$=0.42%; $R_{wp}$=1.70%; $R_p$=1.54%; Gof=4.08.

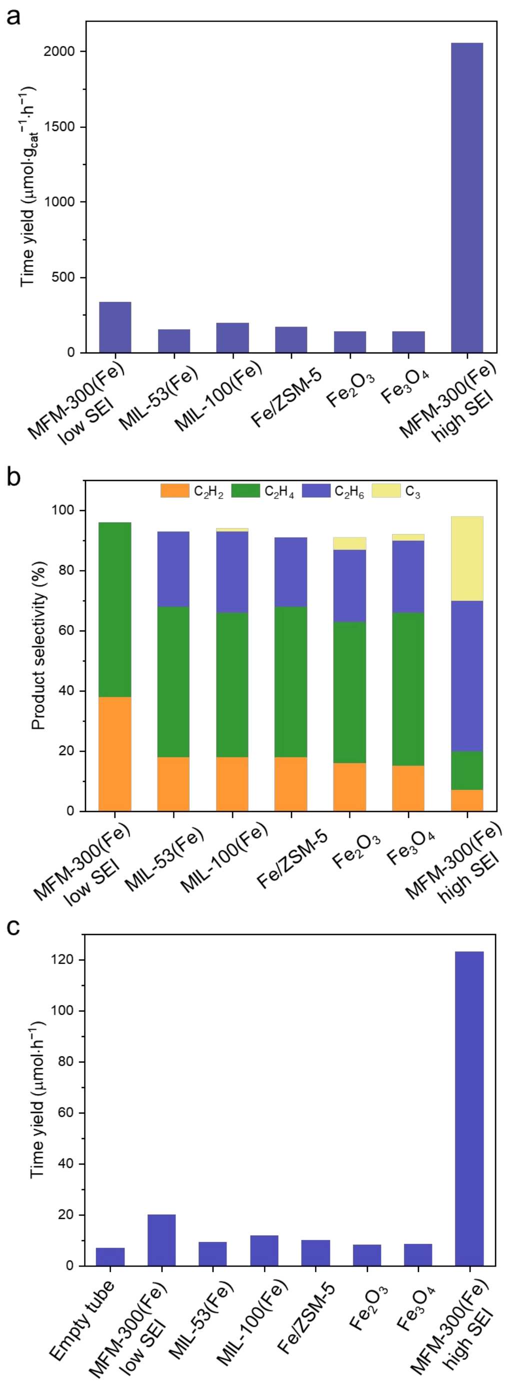


**Figure S14.** Comparison of the catalytic activity (time yield for total $C_{2+}$ products) and product selectivity over different catalysts under activation of NTP. Reaction conditions: specific energy input (SEI) of 8 kJ $L^{-1}$ for MFM-300(Fe) at high SEI, and 2 kJ $L^{-1}$ in other experiments; 2% $CH_4$ in He for MFM-300(Fe) at high SEI and 1% $CH_4$ in He for other experiments, with a total flow rate for the gas feed of 60 mL $min^{-1}$; 60 mg of catalyst, 25 °C and 1 atm.

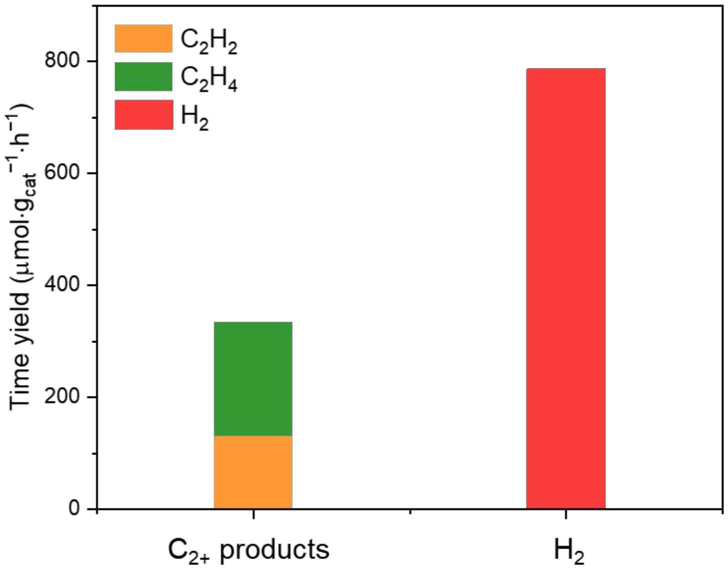


**Figure S15.** Time yield of $C_{2+}$ hydrocarbon products and $H_2$ over MFM-300(Fe) under activation of NTP. Reaction conditions: specific energy input of 2 kJ $L^{-1}$, 1% $CH_4$ in He as gas feed at a total flow rate of 60 mL $min^{-1}$, 60 mg of catalyst, 25 °C and 1 atm.

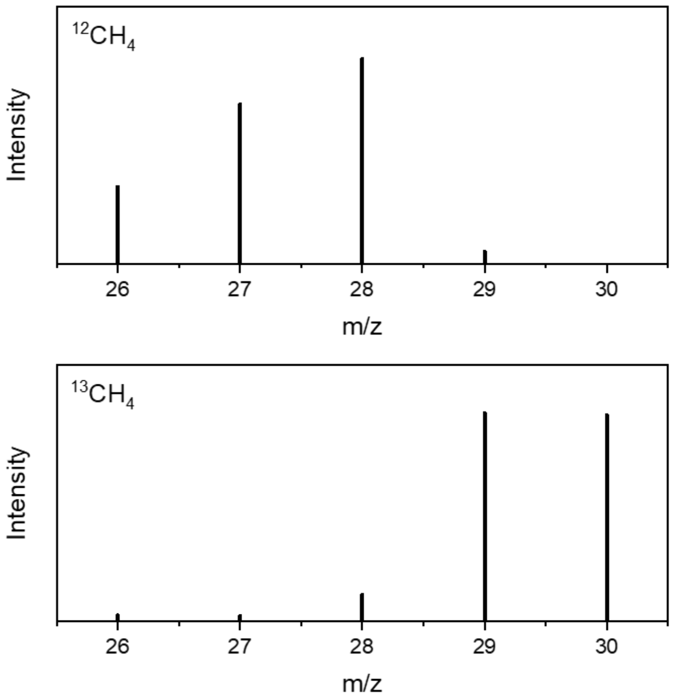


**Figure S16.** Mass spectra of $C_2$ products produced over MFM-300(Fe) using $^{12}CH_4$ and $^{13}CH_4$ as reactants.

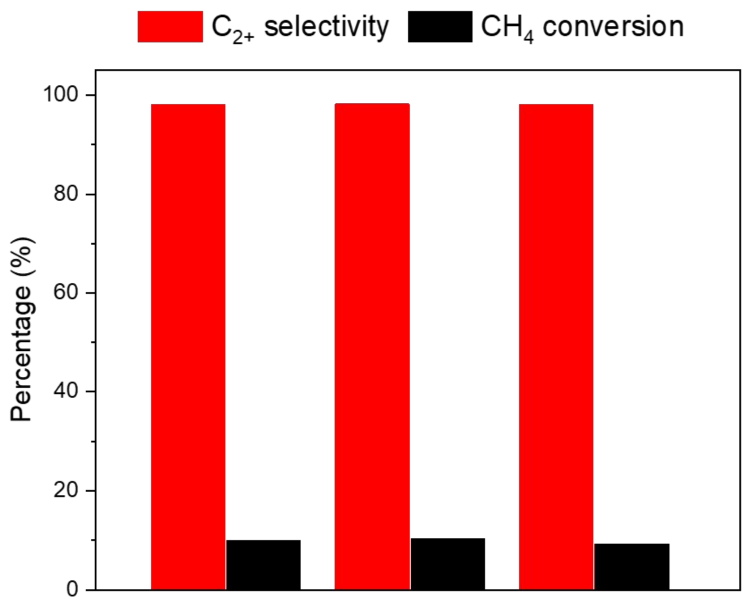


**Figure S17.** Comparison of $C_{2+}$ selectivity and $CH_4$ conversion over 3 cycles performed at a high specific energy input of 8 kJ $L^{-1}$ with a $CH_4$ conversion of ~10%; 2% $CH_4$ in He with a total flow rate of 60 mL $min^{-1}$ as feed gas, 60 mg of catalyst, 25 °C and 1 atm.

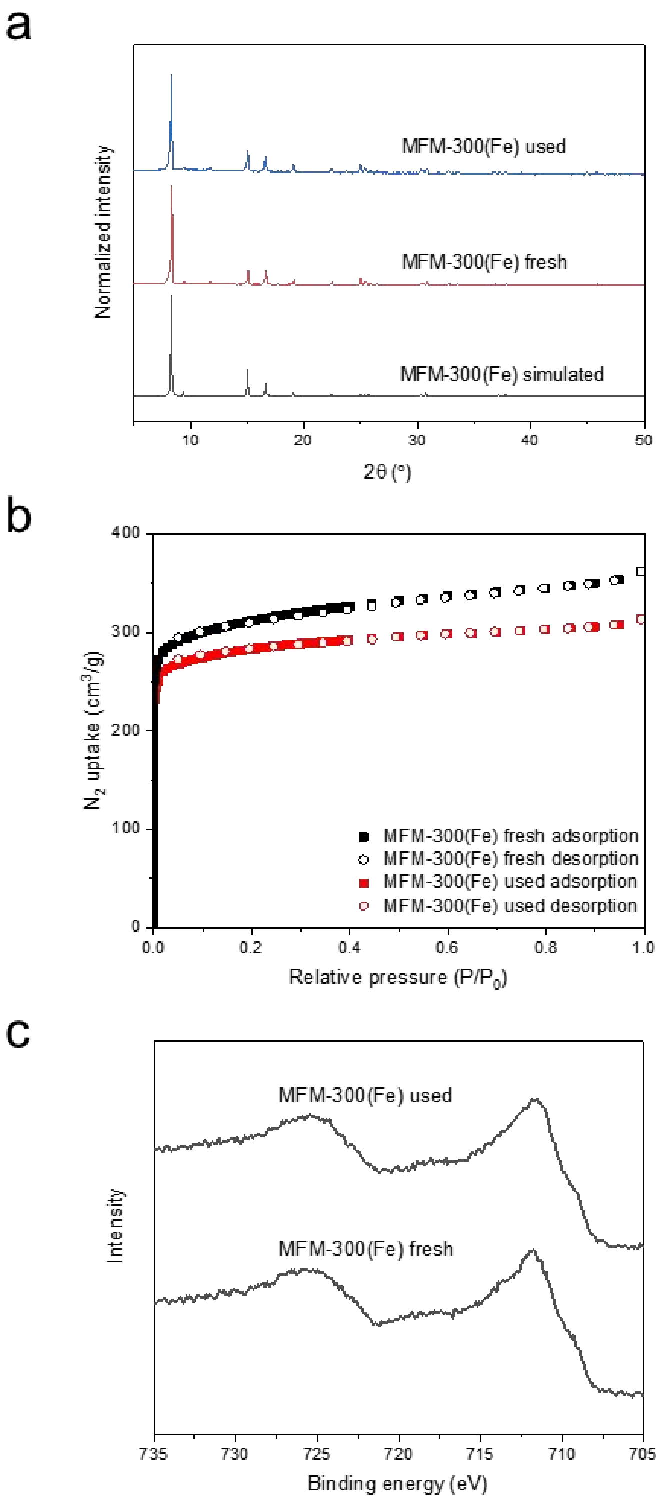


**Figure S18.** Characterisation of used MFM-300(Fe). (a) PXRD patterns showing retention of crystallinity. (b) $N_2$ adsorption isotherms showing retention of porosity. (c) Fe 2p XPS spectra.

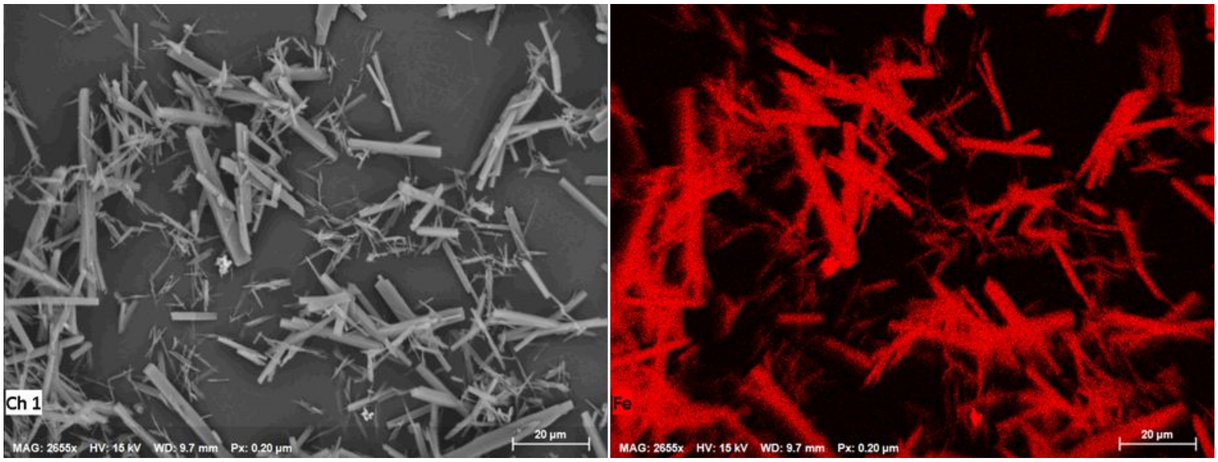


**Figure S19.** SEM image and EDX mapping of the used MFM-300(Fe).

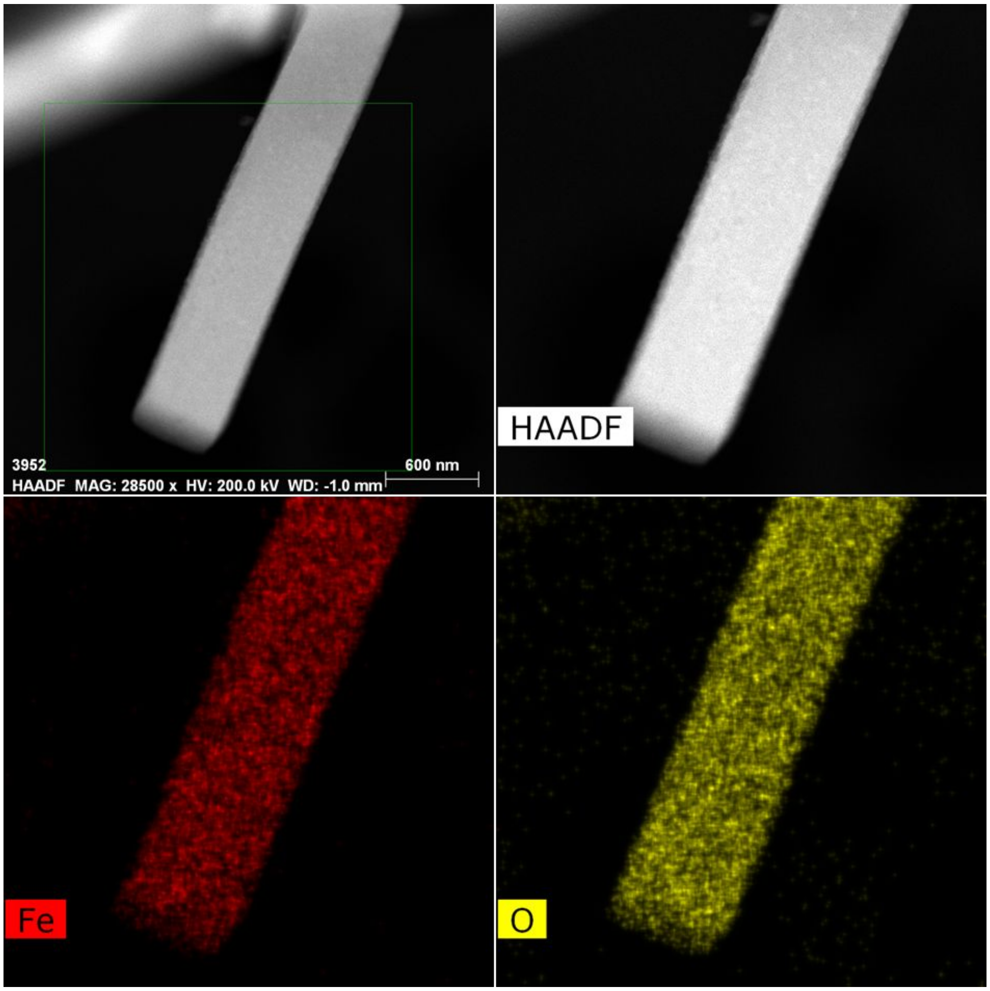


**Figure S20.** HAADF-STEM images and EDX mapping of the used MFM-300(Fe).

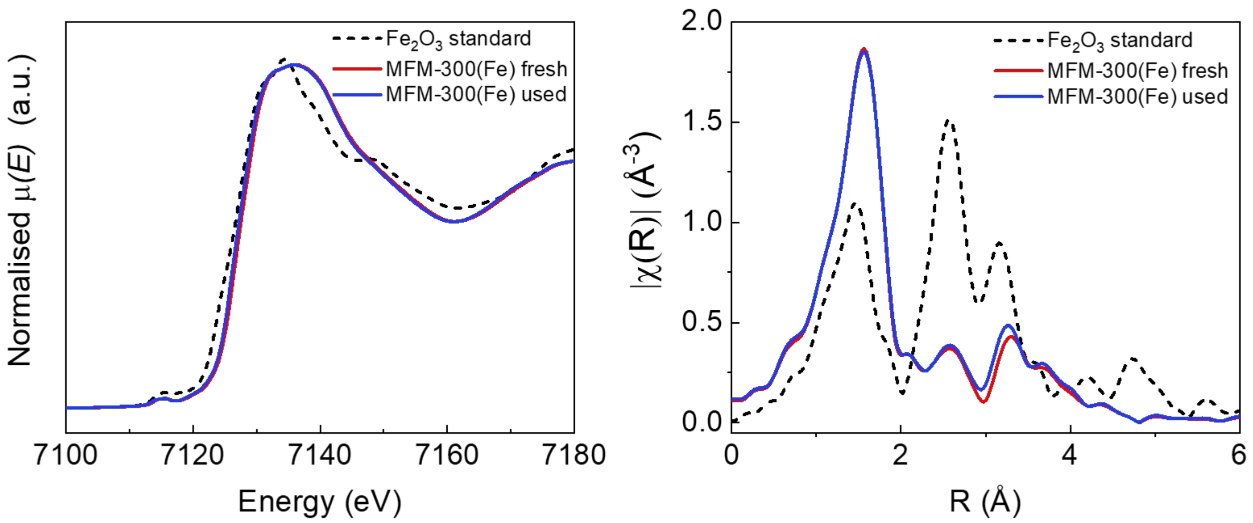


**Figure S21.** Normalised Fe K-edge XANES spectrum of used MFM-300(Fe) and standard XANES spectrum of $Fe_2O_3$ reference (left). Plot of non-phase corrected Fourier transformed Fe K-edge EXAFS data for used MFM-300(Fe), shown against a $Fe_2O_3$ standard reference (right).

**Table S2.** Fe K-edge EXAFS fitting parameters of fresh and used MFM-300(Fe).

| Sample | Absorber - Scatterer | $N$ | $R$ (Å) | $\sigma^2$ (Å$^2$) | $E_0$ (eV) | $R_{\text{factor}}$ |
|---|---|---|---|---|---|---|
| MFM-300(Fe) fresh | Fe−O | 6.10(5) | 1.996(5) | 0.0062(6) | 2.22(1) | 0.015 |
| MFM-300(Fe) used | Fe−O | 6.17(6) | 1.999(6) | 0.0065(2) | 2.61(2) | 0.020 |

Note: Fitting parameters: $S_0^2 = 0.86$; fit range $3 < k(Å−2) < 10.5$, $1 < R(Å) < 3$; number of independent points = 9.3.

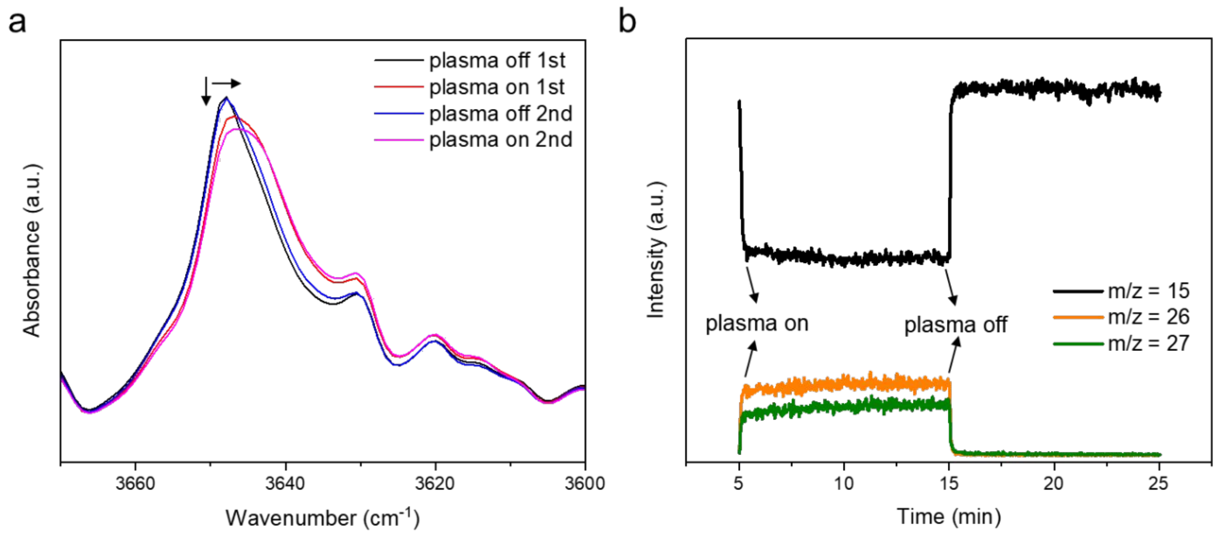


**Figure S22.** (a) *In situ* DRIFT spectra of MFM-300(Fe) in NTP-assisted $CH_4$ conversion as a function of plasma on and off, showing the red shift of the υ(O−H) stretching mode of the $\mu_2$−OH in MFM-300(Fe). All the DRIFT spectra were recorded at a resolution of 4 $cm^{-1}$, with the spectrum of KBr subtracted as the background. (b) Mass spectrometric (MS) signals measured at the exit of the DRIFTS cell during NTP-assisted $CH_4$ conversion (plasma is turned on at 5 mins, and turned off at 15 mins).

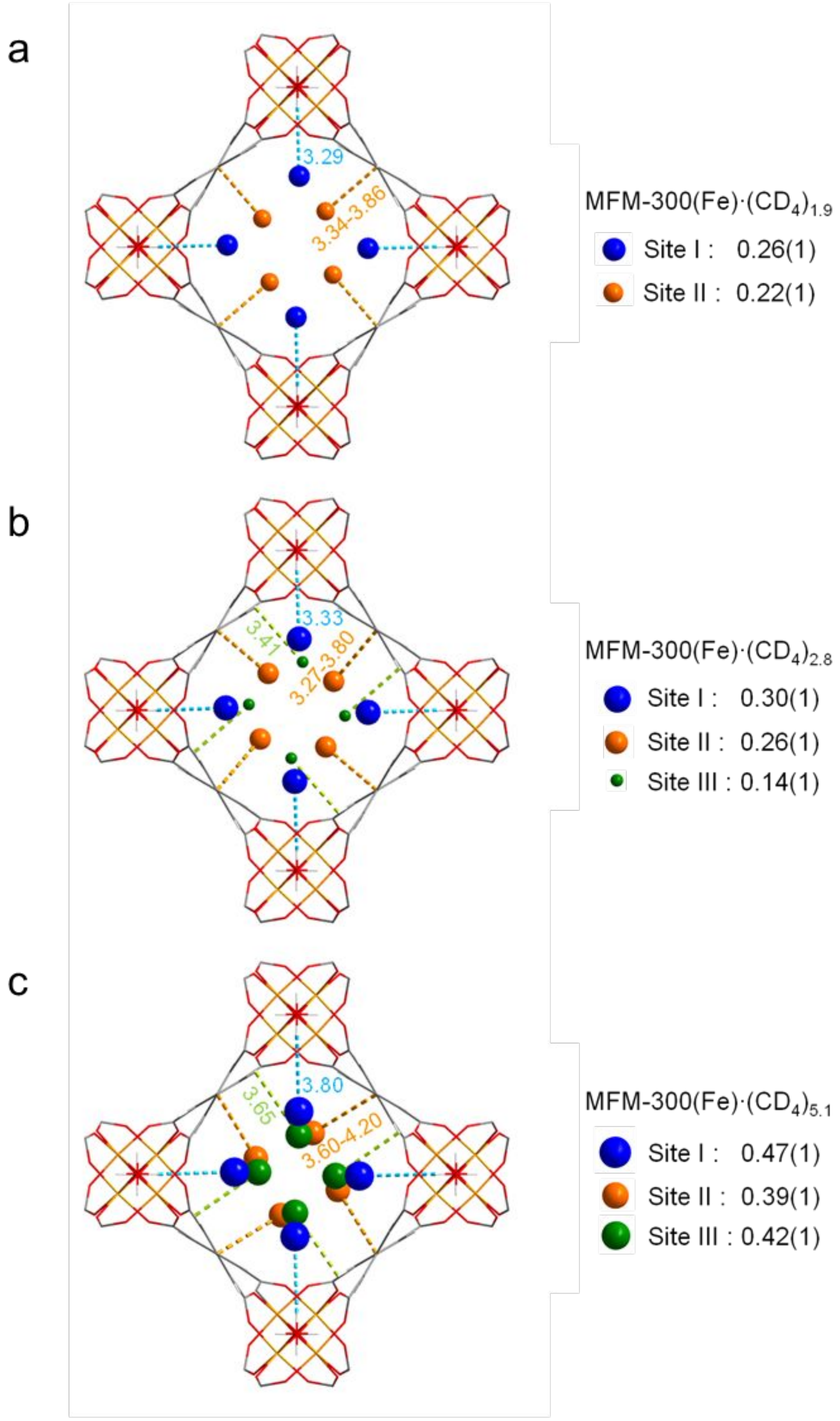


**Figure S23.** Views of the binding sites for $CD_4$ molecules in (a) MFM-300(Fe)·$(CD_4)_{1.9}$, (b) MFM-300(Fe)·$(CD_4)_{2.8}$ and (c) MFM-300(Fe)·$(CD_4)_{5.1}$. All structures were derived from Rietveld refinements of NPD data collected at 7 K (C, grey; O, red; Fe, light orange; H, white; $CD_4$ molecules are coloured according to the scheme: Site I, blue; Site II, orange; Site III, green).

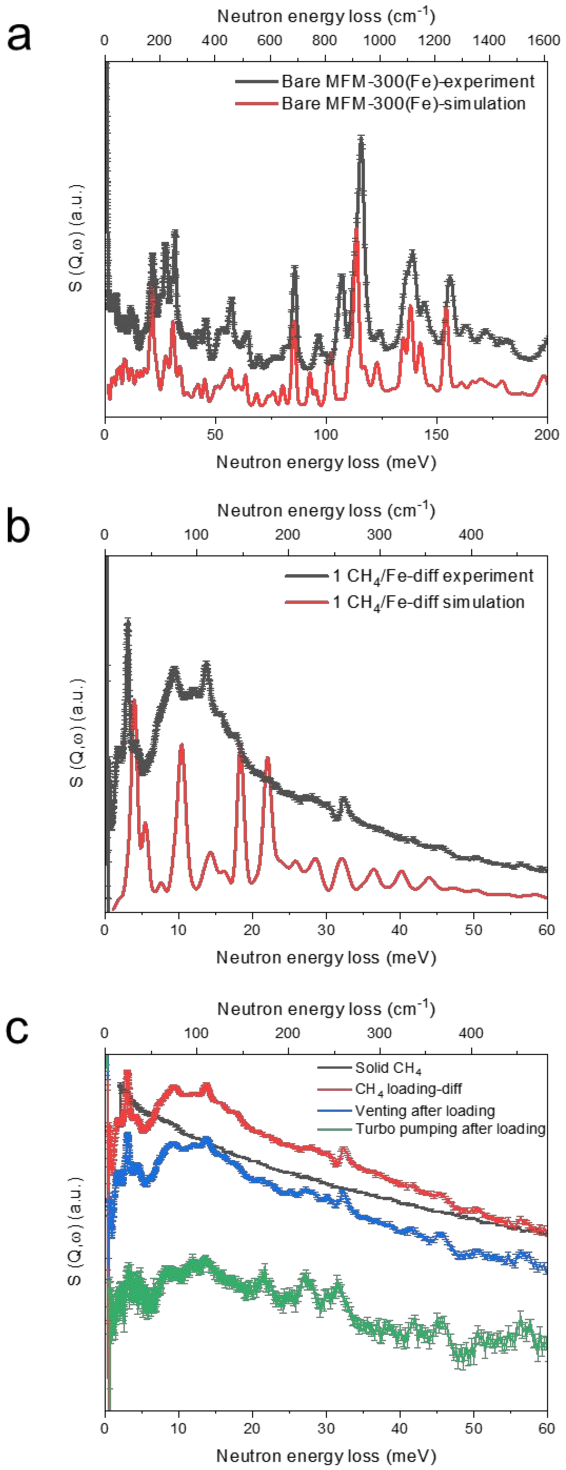


**Figure S24.** (a) Comparison of experimental and simulated INS spectra for bare MFM-300(Fe). (b) Comparison of experimental and simulated difference INS spectra for $CH_4$ loaded MFM-300(Fe). (c) Comparison of the difference INS spectra for $CH_4$ loaded MFM-300(Fe), the difference INS spectra upon evacuating under dynamic vacuum with a turbo pump, and the INS spectra for solid $CH_4$.

The INS spectra in Figure S24a show excellent agreement between simulated and measured bare MFM-300(Fe), confirming that the sample is clean and the structural model for simulation is correct. This affords the assignment of INS peaks. The strongest peaks in the low-energy region (<40 meV) are all due to $CH_4$ libration, including translational and rotational motions. In Figure S24c, the difference spectra show sharp peaks for adsorbed $CH_4$ that are very different from solid $CH_4$. The peaks are sharp because the $CH_4$ are pinned to the adsorption site with very little recoil, whereas in solid $CH_4$ the intermolecular interaction is much weaker and the incident neutrons cause significant recoil. In order to detect the presence of bound $CH_4$, the sample cell was evacuated under dynamic vacuum with a turbo pump. The remaining $CH_4$ appears to be in a similar adsorption status (Figure S24c, blue line). The sample cell was then evacuated with a turbo pump for another 45 minutes. Surprisingly, this process did not remove all $CH_4$, with some INS signal for $CH_4$ still detected (Figure S24c, green line), confirming that some $CH_4$ molecules are bound to MFM-300(Fe). Interestingly, the integrated intensity of the entire spectrum (including both elastic and inelastic scattering), which scales roughly with the amount of $CH_4$, has a ratio of 1:0.66:0.26 among the three curves in Figure S24c (red, blue and green curves). The initial loading was about 4 mmol $g^{-1}$ of $CH_4$ (1 $CH_4$ per Fe site). Therefore, there was ~1 mmol $g^{-1}$ of $CH_4$ retained within the pores after desorption, consistent with the $CH_4$ isotherm data.

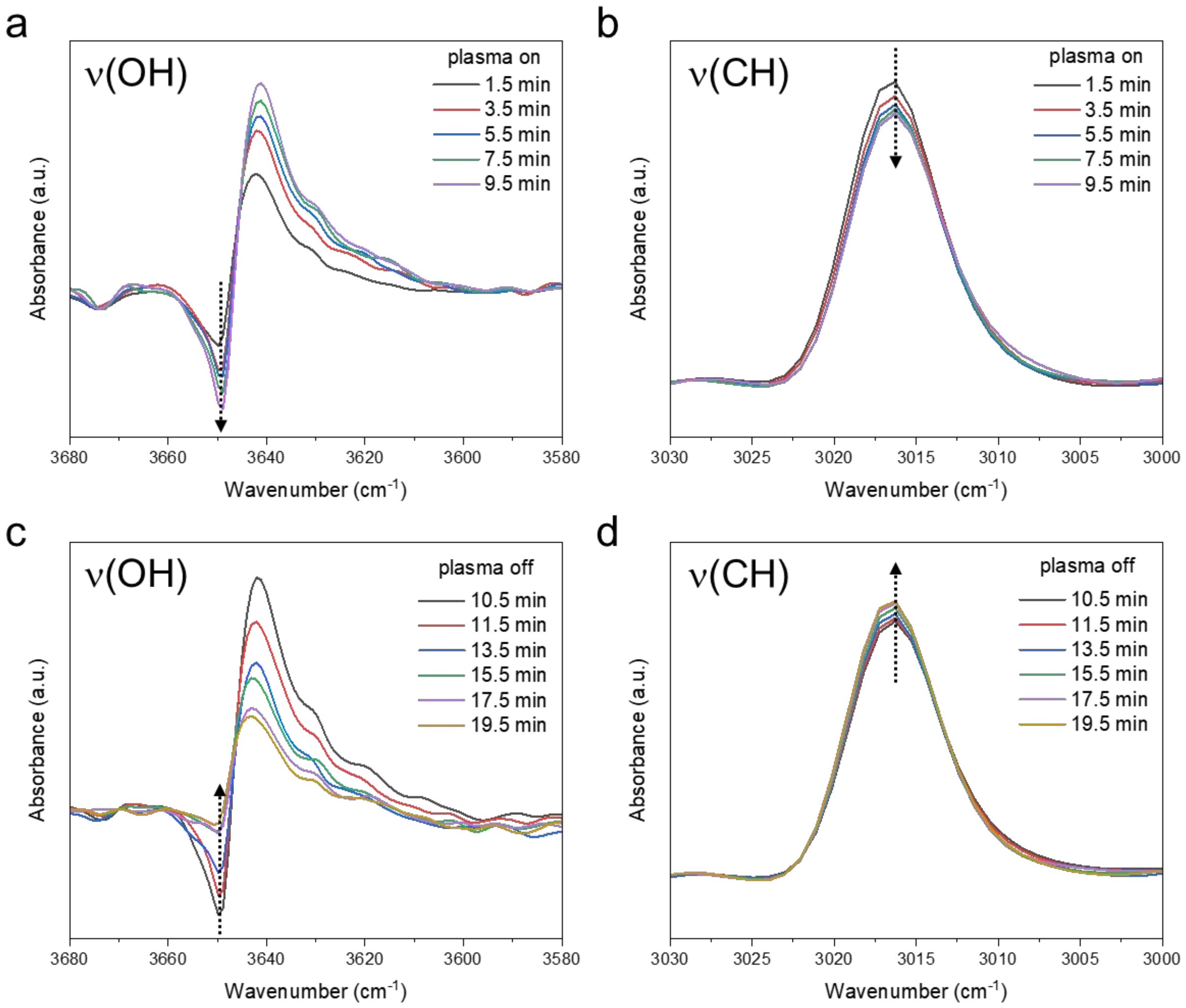


**Figure S25.** *In situ* DRIFT spectra showing (a, c) the ν(OH) stretching vibration in MFM-300(Fe), and (b, d) the ν(CH) stretch of $CH_4$ in NTP-assisted $CH_4$ conversion reactions as a function of plasma on-off and reaction time. The correlation between relative intensities of the ν(OH) peak at 3649 $cm^{-1}$ and ν(CH) peak at 3016 $cm^{-1}$ is shown in Figure 3b.

Surface −OH species that are relevant to the activation of $CH_4$ and stabilisation of intermediates will show changes at a similar rate to that observed for the gas phase reactants. The relative correlation between ν(OH) at 3649 $cm^{-1}$ (MOF) and ν(CH) at 3016 $cm^{-1}$ as a function of ToS during plasma on-off is shown in Figure 3b and Figure S25. Interestingly, it is observed that the rate of decrease/increase of ν(CH) from $CH_4$ (g/ads.) matches very closely with that of surface ν(OH) upon switching plasma on/off (Figure 3b and Figure S25). This close correlation between the two species indicates that $CH_4$ molecules are activated and converted at the $\mu_2$−OH sites.

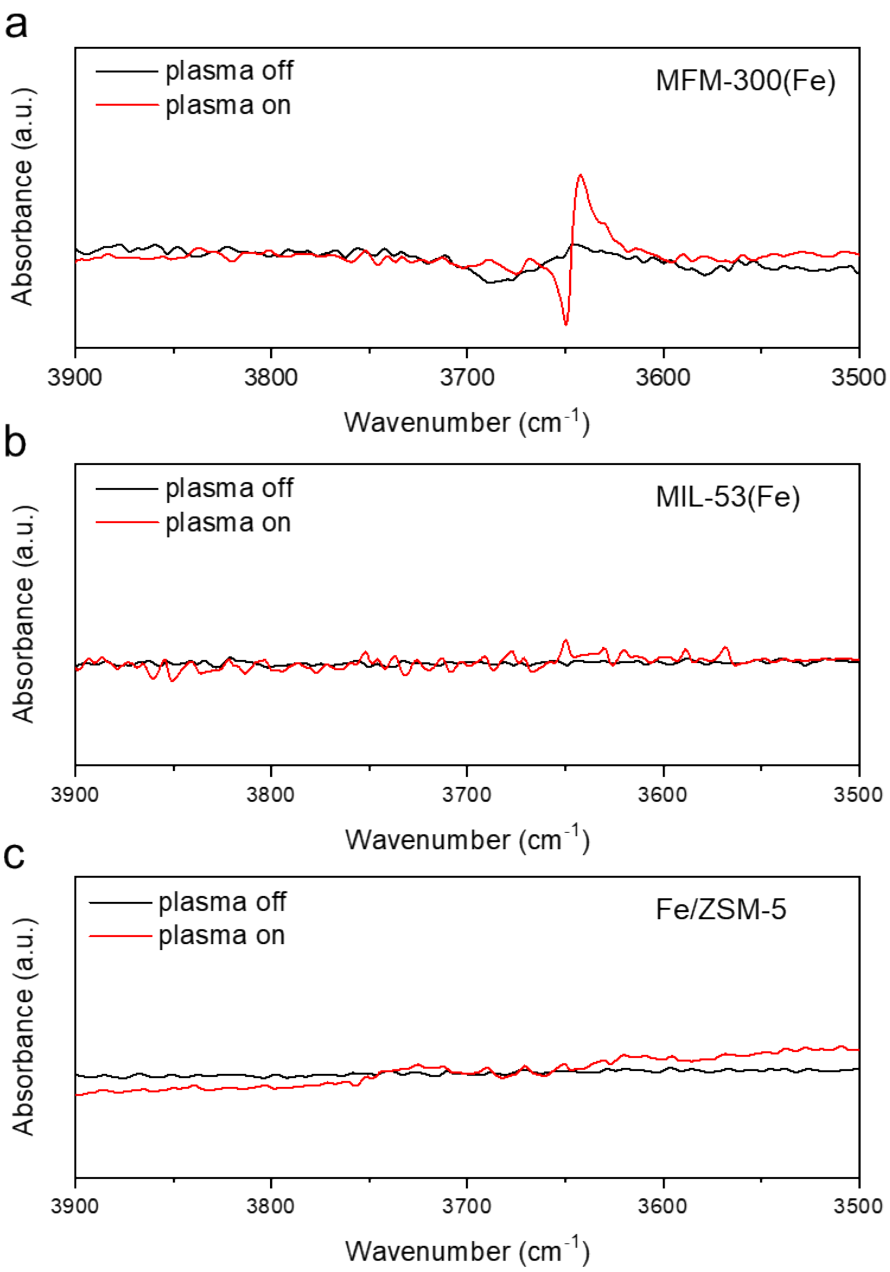


**Figure S26.** *In situ* DRIFT spectra of catalysts [top to bottom: MFM-300(Fe), MIL-53(Fe) and Fe/ZSM-5] in NTP-assisted $CH_4$ conversion reaction as a function of plasma off and on. $CH_4$ was flowing for both plasma off and on. All the DRIFT spectra were recorded at a resolution of 4 $cm^{-1}$, and the spectra of bare catalysts have been subtracted as the background.

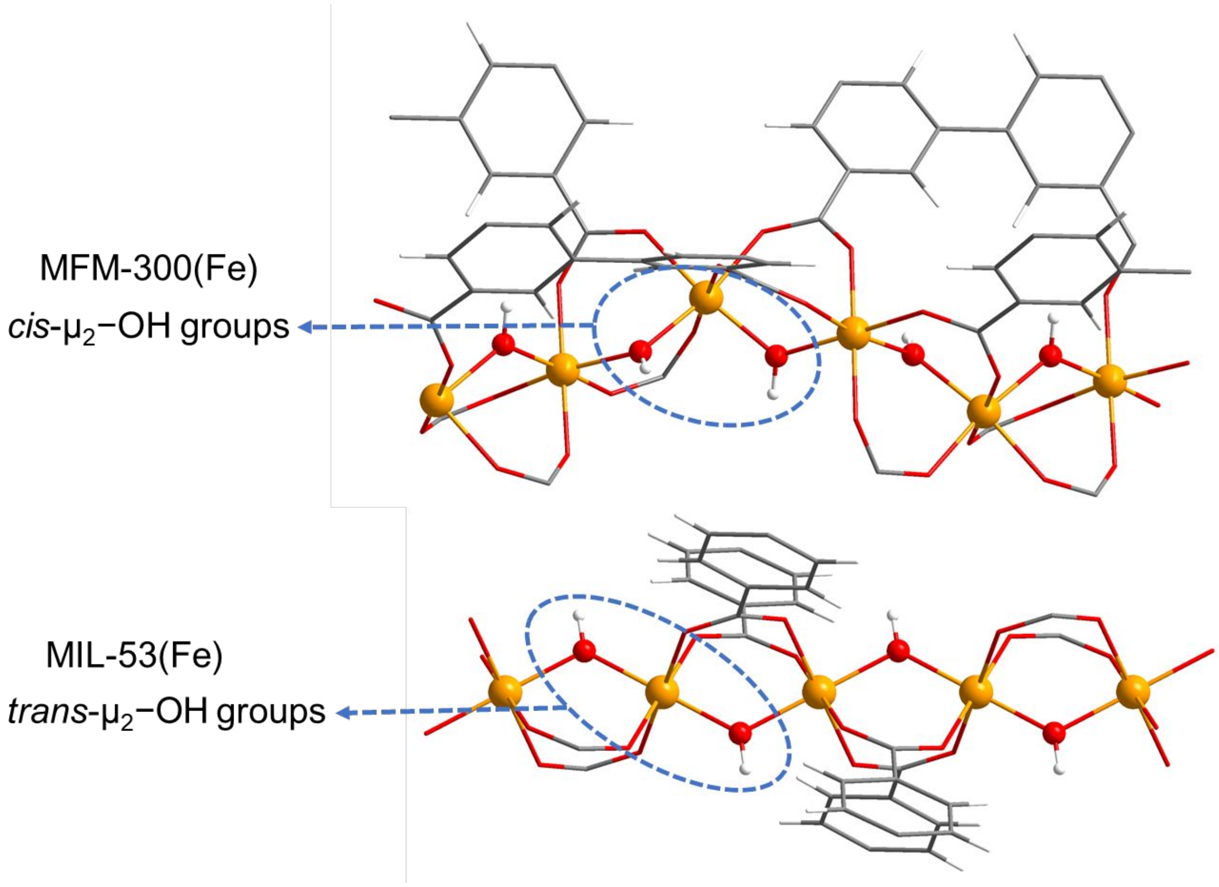


**Figure S27.** Views of the structures for MFM-300(Fe) (top) and MIL-53(Fe) (bottom). (C, grey; O, red; Fe, light orange; H, white)

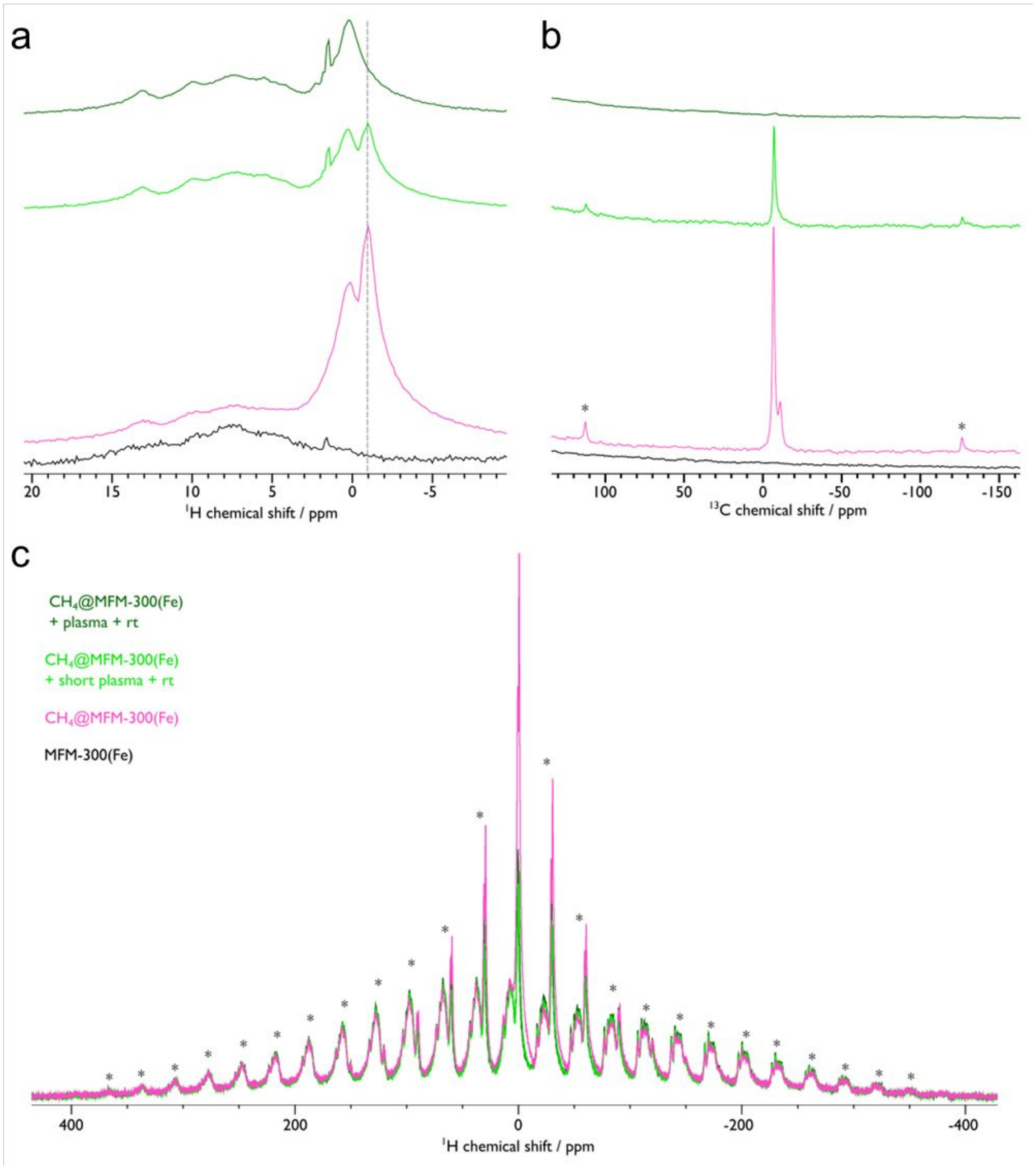


**Figure S28.** Solid-state magic-angle spinning (a, c) $^1$H and (b) $^{13}$C NMR spectra of bare MFM-300(Fe) (black), $CH_4$-loaded MFM-300(Fe) (pink), $CH_4$-loaded MFM-300(Fe) after a short plasma treatment and then room temperature equilibration (light green), and $CH_4$-loaded MFM-300(Fe) after a long plasma treatment then room temperature equilibration (dark green). Asterisks denote spinning side bands and the vertical dashed grey line in (a) highlights a $^1$H resonance from weaker-bound $CH_4$ species. The $CH_4$ used for solid-state NMR experiments is $^{13}$C labelled.

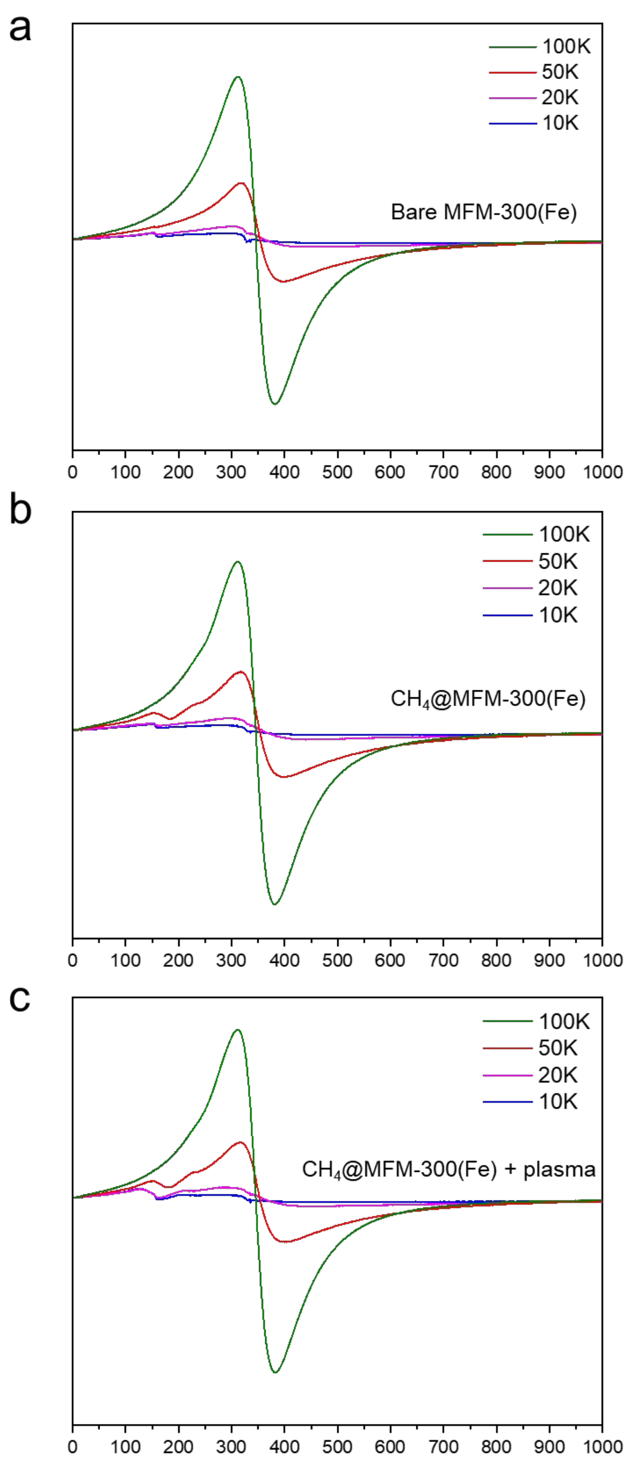


**Figure S29.** X-band (9.4 GHz) EPR spectra recorded at 100-10 K for (a) bare MFM-300(Fe), (b) $CH_4$-loaded MFM-300(Fe), and (c) $CH_4$-loaded MFM-300(Fe) after plasma treatment.

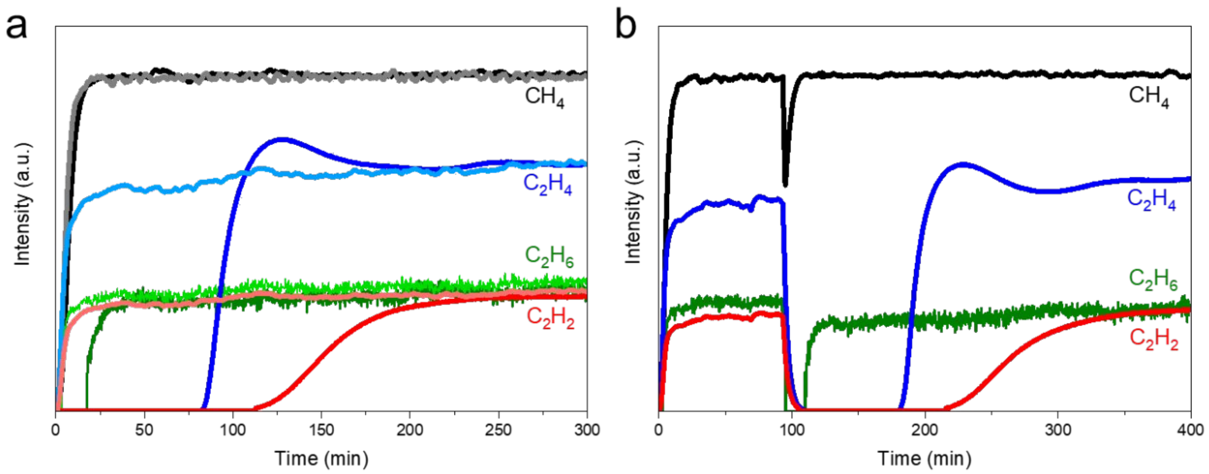


**Figure S30.** Separation of $C_{2+}$ products produced at a specific energy input of 8 kJ $L^{-1}$ from unreacted $CH_4$ using a packed bed of ZSM-5. (a) Output from the reaction reactor fixed bed-1 (line 1 in Figure S1) without any separation: light grey ($CH_4$), light blue ($C_2H_4$), light green ($C_2H_6$) and light red ($C_2H_2$). Passing the mixture of products through fixed bed-2 of ZSM-5 (line 2 in Figure S1): black ($CH_4$), blue ($C_2H_4$), green ($C_2H_6$) and red ($C_2H_2$). (b) Switching from line 1 to line 2 after 90 mins of reaction: black ($CH_4$), blue ($C_2H_4$), green ($C_2H_6$) and red ($C_2H_2$).

**Table S3.** Comparison of the reported catalysts for $CH_4$ conversion to $C_{2+}$ products.

| Entry | Catalysts | Selectivity (%) | Main products | Time yield ($\mu mol_{C2+} \cdot g_{cat}^{-1} \cdot h^{-1}$) | Reaction conditions | Ref |
|---|---|---|---|---|---|---|
| 1 | MFM-300(Fe) | 96 | $C_2H_2$+$C_2H_4$ | 334 | plasma flow reactor at specific energy input of 2 kJ $L^{-1}$ | This work |
| 2 | MFM-300(Fe) | >98 | $C_2H_2$+$C_2H_4$+$C_2H_6$+ $C_3H_6$+$C_3H_8$ | 2056 | plasma flow reactor at specific energy input of 8 kJ $L^{-1}$ | This work |
| 3 | Ag–HPW/$TiO_2$ | >90 | $C_2H_6$+$C_3H_8$ | 24.2 | photochemical looping | 11 |
| 4 | Pt/HGTS | 90 | $C_2H_6$ | ~2 | photochemical batch reactor | 12 |
| 5 | ZnO−AuPd | 96 | $C_2H_4$+$C_2H_6$+$C_3H_6$+$C_3H_8$ | 32 | photochemical batch reactor | 13 |
| 6 | Au/m-ZnO | not mentioned | $C_2H_6$ | 13 | photochemical batch reactor | 14 |
| 7 | $Cu_{0.1}Pt_{0.5}$/PC-50 | 60 | $C_2H_4$+$C_2H_6$ | 68 | photochemical flow reactor | 15 |
| 8 | Au/$TiO_2$ | >95 | $C_2H_6$ | 81.7 | photochemical flow reactor | 16 |
| 9 | Pt/$CeO_2$ | 60 | $C_2H_2$+$C_2H_4$+ $C_2H_6$ | ~464 | thermal (780-910 °C) | 17 |
| 10 | $BaCe_{0.8}Gd_{0.2}O_{3-\delta}$ | >80 | $C_2H_4$+$C_2H_6$+$C_3H_6$+$C_3H_8$ | not mentioned | thermal (800 °C) | 18 |
| 11 | Fe©$SiO_2$ | 99 | $C_2H_4$+benzene+naphthalene | 118000 | thermal (1090 °C) | 19 |
| 12 | $BaTiO_3$ | 55 | $C_2H_2$+$C_2H_4$+$C_2H_6$+ $C_3H_6$+$C_3H_8$+$C_{4+}$ | not mentioned | plasma flow reactor | 20 |
| 13 | Ag/$SiO_2$ | 36 | $C_2H_4$+$C_2H_6$+$C_3H_6$+$C_3H_8$ | not mentioned | plasma flow reactor | 21 |
| 14 | Pt/γ-$Al_2O_3$ | 70% | $C_2H_2$+$C_2H_4$+$C_2H_6$+ $C_3H_6$+$C_3H_8$ | not mentioned | plasma flow reactor | 22 |
| 15 | Zn-ZSM-5 | >99 | $C_2H_6$ | 4.9 | photochemical batch reactor | 23 |
| 16 | Ga-ETS-10 | >70% | $C_2H_6$ | 10.9 | photochemical batch reactor | 24 |
| 17 | Pt/$TiO_2$ | 62 | $C_2H_6$ | 53 | photochemical batch reactor | 25 |
| 18 | $Pd_1$/$TiO_2$ | 94 | $C_2H_6$ | 910 | photochemical batch reactor | 26 |

**Cartesian coordinates of the corresponding structure of the DFT calculation**

$CH_4$

| | | | |
|---|---|---|---|
| C | -0.00000200 | 0.00001400 | -0.00000200 |
| H | 0.06712700 | -1.09742100 | 0.17309200 |
| H | 0.92122900 | 0.35110200 | -0.51656500 |
| H | -0.88802800 | 0.22205900 | -0.63311500 |
| H | -0.10031600 | 0.52417600 | 0.97659900 |

MFM-300(Fe)

| | | | |
|---|---|---|---|
| O | -3.78229300 | 2.49193400 | 0.23720100 |
| O | -2.45453800 | 1.15394100 | 1.28721100 |
| C | -2.99898500 | 2.23073700 | 1.13049000 |
| C | -2.68555100 | 3.29524300 | 2.09150500 |
| C | -1.43826900 | 3.29523900 | 2.76940800 |
| H | -0.73842600 | 2.48447300 | 2.59684900 |
| O | 3.41334600 | -1.37113000 | -0.43773700 |
| O | 5.01661900 | -0.03315300 | 0.10529000 |
| C | 4.58900600 | -1.10993900 | -0.26631900 |
| C | 5.56588600 | -2.17446300 | -0.52601500 |
| C | 6.81318400 | -2.17444500 | 0.15185900 |
| H | 7.04952700 | -1.36746100 | 0.83650900 |
| O | -1.10688500 | -1.45417400 | 1.59677100 |
| O | 0.99758200 | -1.74269800 | 1.21770700 |
| C | -0.00267800 | -1.86498000 | 1.89963300 |
| C | 0.13423900 | -2.55966900 | 3.18562400 |
| C | 1.38152100 | -2.55967300 | 3.86352700 |
| H | 2.24060300 | -2.08421600 | 3.40206700 |
| O | 3.78239500 | 2.49186900 | -0.23721000 |
| O | 2.45462400 | 1.15387100 | -1.28719900 |
| C | 2.99908300 | 2.23066700 | -1.13049500 |
| C | 2.68557800 | 3.29516100 | -2.09157700 |
| C | 1.43829700 | 3.29516400 | -2.76948100 |
| H | 0.73844100 | 2.48441800 | -2.59687800 |
| O | -3.41330500 | -1.37106700 | 0.43780000 |
| O | -5.01658000 | -0.03309000 | -0.10528000 |
| C | -4.58892800 | -1.10984300 | 0.26640700 |
| C | -5.56597100 | -2.17433000 | 0.52609100 |
| C | -6.81323200 | -2.17435400 | -0.15185100 |
| H | -7.04955300 | -1.36737700 | -0.83651700 |
| O | 1.10683700 | -1.45422200 | -1.59675400 |
| O | -0.99763100 | -1.74270300 | -1.21768400 |
| C | 0.00262200 | -1.86501600 | -1.89960800 |

| | | | |
|---|---|---|---|
| C | -0.13421800 | -2.55972700 | -3.18553800 |
| C | -1.38149900 | -2.55972300 | -3.86344200 |
| H | -2.24058300 | -2.08425100 | -3.40200100 |
| C | 6.19182700 | -4.25586500 | -1.67770100 |
| C | 7.43911100 | -4.25586600 | -0.99980200 |
| H | 8.15455600 | -5.05255500 | -1.17917800 |
| C | -3.31148200 | 5.37665900 | 3.24317400 |
| C | -2.06420000 | 5.37665500 | 3.92107700 |
| H | -1.82648300 | 6.17152300 | 4.62142600 |
| C | -0.85970500 | -3.87044400 | 5.01438500 |
| C | 0.38757700 | -3.87044800 | 5.69228800 |
| H | 0.48520300 | -4.37162000 | 6.65039000 |
| C | -6.19192300 | -4.25573200 | 1.67777500 |
| C | -7.43920400 | -4.25572800 | 0.99987100 |
| H | -8.15465900 | -5.05240100 | 1.17927700 |
| C | 3.31154300 | 5.37654500 | -3.24328500 |
| C | 2.06426200 | 5.37654800 | -3.92118900 |
| H | 1.82656700 | 6.17141000 | -4.62155000 |
| C | 0.85970500 | -3.87055200 | -5.01427400 |
| C | -0.38757600 | -3.87054900 | -5.69217800 |
| H | -0.48522000 | -4.37173400 | -6.65027100 |
| Fe | -4.32871000 | 1.34575500 | -1.45890300 |
| Fe | 1.69288700 | -0.22492500 | 0.02631800 |
| Fe | -1.69285900 | -0.22495700 | -0.02628400 |
| Fe | 4.32878700 | 1.34572800 | 1.45892300 |
| O | -2.60256900 | 0.56042200 | -1.49360000 |
| H | -2.19705300 | 0.34783600 | -2.35376400 |
| O | 2.60266900 | 0.56035700 | 1.49361600 |
| H | 2.19697500 | 0.34812900 | 2.35390900 |
| O | -0.00001100 | 0.62996100 | -0.00000800 |
| H | -0.00011400 | 1.60288100 | 0.00058400 |
| C | -3.62259600 | 4.33595300 | 2.32814900 |
| C | -1.12706700 | 4.33594600 | 3.68448100 |
| H | -4.57101200 | 4.32648600 | 1.80266300 |
| C | 5.25478800 | -3.21514700 | -1.44106900 |
| C | 7.75031100 | -3.21516300 | -0.08472500 |
| H | 4.29566800 | -3.20611500 | -1.94628500 |
| C | -0.98690100 | -3.21505500 | 3.76076600 |
| C | 1.50871700 | -3.21506200 | 5.11714600 |
| H | -1.93510700 | -3.19665000 | 3.23587000 |
| C | 3.62272900 | 4.33585100 | -2.32819300 |
| C | 1.12711100 | 4.33585800 | -3.68457300 |
| H | 4.57114200 | 4.32637700 | -1.80271300 |
| C | -5.25476500 | -3.21505200 | 1.44114500 |
| C | -7.75039000 | -3.21503400 | 0.08477900 |
| H | -4.29563800 | -3.20604200 | 1.94633600 |

| | | | |
|---|---|---|---|
| C | 0.98682400 | -3.21514200 | -3.76071600 |
| C | -1.50870600 | -3.21513400 | -5.11704800 |
| H | 1.93503400 | -3.19674600 | -3.23581600 |
| H | 5.95453400 | -5.04782900 | -2.38085200 |
| H | -4.03194300 | 6.16736800 | 3.42685900 |
| H | -1.71612500 | -4.37469700 | 5.45067100 |
| H | -5.95466200 | -5.04771700 | 2.38091500 |
| H | 4.03199700 | 6.16725500 | -3.42700700 |
| H | 1.71613500 | -4.37481900 | -5.45052300 |
| H | -0.17247000 | 4.33356900 | 4.20153400 |
| H | 8.70481400 | -3.20936500 | 0.43176600 |
| H | 2.46467300 | -3.21873300 | 5.63148100 |
| H | 0.17251300 | 4.33348400 | -4.20162300 |
| H | -8.70488800 | -3.20923100 | -0.43172200 |
| H | -2.46466000 | -3.21879900 | -5.63138600 |
| O | -4.19903300 | 2.58770800 | -2.94238500 |
| O | -5.96081100 | 2.42622800 | -1.69592200 |
| H | -5.86334300 | 3.78427100 | -3.25424500 |
| C | -5.38425900 | 2.99251200 | -2.66974200 |
| O | 4.19876400 | 2.58815500 | 2.94201100 |
| O | 5.96074600 | 2.42631000 | 1.69590400 |
| H | 5.86281200 | 3.78522300 | 3.25343600 |
| C | 5.38394700 | 2.99303900 | 2.66932700 |

MFM-300(Fe)-$CH_4$

| | | | |
|---|---|---|---|
| O | 3.80104800 | 2.32404200 | -0.34578900 |
| O | 2.44441100 | 0.97662600 | -1.34568900 |
| C | 3.01012600 | 2.04743800 | -1.22761700 |
| C | 2.71458200 | 3.08418800 | -2.22426500 |
| C | 1.46626100 | 3.08465800 | -2.89885400 |
| H | 0.75052600 | 2.29430300 | -2.69659600 |
| O | -3.46607000 | -1.37232600 | 0.47930800 |
| O | -5.04473300 | -0.02304700 | -0.10600800 |
| C | -4.63698300 | -1.09436600 | 0.30170000 |
| C | -5.63356400 | -2.13010100 | 0.60041700 |
| C | -6.88265200 | -2.12916200 | -0.07418200 |
| H | -7.10530800 | -1.34199200 | -0.78579300 |
| O | 1.04556300 | -1.61387400 | -1.56156000 |
| O | -1.06299100 | -1.84801600 | -1.16780200 |
| C | -0.06729800 | -2.01331500 | -1.84737300 |
| C | -0.22149500 | -2.74931400 | -3.10819600 |
| C | -1.47058500 | -2.74847600 | -3.78279100 |
| H | -2.31891700 | -2.24063900 | -3.33616000 |
| O | -3.76077400 | 2.48786600 | 0.14588300 |

| | | | |
|---|---|---|---|
| O | -2.45603400 | 1.16139500 | 1.23855100 |
| C | -2.97994200 | 2.24262300 | 1.04574500 |
| C | -2.64287500 | 3.33283800 | 1.96844000 |
| C | -1.39473200 | 3.33352500 | 2.64423600 |
| H | -0.71053600 | 2.50253400 | 2.49737600 |
| O | 3.35660200 | -1.53566200 | -0.41149400 |
| O | 4.98716800 | -0.21116000 | 0.08117300 |
| C | 4.53755600 | -1.29154300 | -0.25198400 |
| C | 5.49304600 | -2.38323200 | -0.47689200 |
| C | 6.74203400 | -2.38416500 | 0.19780700 |
| H | 6.99608900 | -1.55871100 | 0.85346900 |
| O | -1.15814700 | -1.46019200 | 1.63525000 |
| O | 0.93911800 | -1.80259200 | 1.26149600 |
| C | -0.06121900 | -1.88170200 | 1.94954600 |
| C | 0.06593300 | -2.53400800 | 3.25843500 |
| C | 1.31502300 | -2.53484700 | 3.93303000 |
| H | 2.18179500 | -2.09253400 | 3.45343400 |
| C | -6.29626000 | -4.15779600 | 1.82503000 |
| C | -7.54535000 | -4.15695700 | 1.15043400 |
| H | -8.27562600 | -4.93287700 | 1.35900600 |
| C | 3.37728300 | 5.11168600 | -3.44888800 |
| C | 2.12827900 | 5.11240100 | -4.12364300 |
| H | 1.90385600 | 5.88708900 | -4.85036200 |
| C | 0.74131700 | -4.14165100 | -4.89276100 |
| C | -0.50777300 | -4.14081200 | -5.56735600 |
| H | -0.61794300 | -4.67287900 | -6.50727600 |
| C | 6.07501100 | -4.51505100 | -1.55719400 |
| C | 7.32410100 | -4.51589000 | -0.88259900 |
| H | 8.02347500 | -5.33211300 | -1.03593200 |
| C | -3.22502000 | 5.46507000 | 3.04907500 |
| C | -1.97609300 | 5.46415700 | 3.72378800 |
| H | -1.72086300 | 6.27811900 | 4.39554000 |
| C | -0.94772200 | -3.76120300 | 5.13388200 |
| C | 0.30137000 | -3.76194200 | 5.80847400 |
| H | 0.39210000 | -4.23148300 | 6.78314400 |
| Fe | 4.33019600 | 1.22691100 | 1.38775100 |
| Fe | -1.72515300 | -0.27662800 | -0.02818900 |
| Fe | 1.66015300 | -0.34089000 | 0.01628800 |
| Fe | -4.33437700 | 1.29450700 | -1.50819000 |
| O | 2.58930000 | 0.47696600 | 1.45366300 |
| H | 2.18233100 | 0.30221100 | 2.32165500 |
| O | -2.62394300 | 0.47499200 | -1.51965100 |
| H | -2.22501500 | 0.22517500 | -2.37301400 |
| O | -0.01775000 | 0.54564100 | -0.03338300 |
| H | 0.00520200 | 1.51722500 | -0.07220500 |
| C | 3.67093200 | 4.09734800 | -2.49907900 |

| | | | |
|---|---|---|---|
| C | 1.17120700 | 4.09894600 | -3.84864200 |
| H | 4.62048700 | 4.08763600 | -1.97577400 |
| C | -5.33996400 | -3.14426600 | 1.55031700 |
| C | -7.83904800 | -3.14269100 | 0.20053200 |
| H | -4.37936100 | -3.13647400 | 2.05267400 |
| C | 0.88495600 | -3.44595300 | -3.66293000 |
| C | -1.61422600 | -3.44427300 | -5.01261800 |
| H | 1.83490400 | -3.42777400 | -3.14118900 |
| C | -3.55919000 | 4.39954800 | 2.17152400 |
| C | -1.05969800 | 4.39790700 | 3.52115200 |
| H | -4.50905700 | 4.39047700 | 1.64867200 |
| C | 5.15898100 | -3.44881900 | -1.35453700 |
| C | 7.65816400 | -3.45039900 | -0.00485100 |
| H | 4.19862900 | -3.43852300 | -1.85752100 |
| C | -1.06583900 | -3.14713700 | 3.85861000 |
| C | 1.43324200 | -3.14881500 | 5.20829900 |
| H | -2.01507200 | -3.12844500 | 3.33559000 |
| H | -6.07225700 | -4.92935400 | 2.55470700 |
| H | 4.11232900 | 5.88136600 | -3.66156000 |
| H | 1.58646600 | -4.67734300 | -5.31330200 |
| H | 5.82039100 | -5.32614200 | -2.23186300 |
| H | -3.92949500 | 6.27564400 | 3.20692200 |
| H | -1.81242900 | -4.23321800 | 5.58928600 |
| H | 0.21571600 | 4.09750800 | -4.36302800 |
| H | -8.79479800 | -3.13617500 | -0.31364200 |
| H | -2.57165200 | -3.44704800 | -5.52436400 |
| H | -0.10393200 | 4.39496100 | 4.03557600 |
| H | 8.61413900 | -3.44532400 | 0.50892000 |
| H | 2.39052700 | -3.15333400 | 5.72029800 |
| O | 4.22904800 | 2.52170900 | 2.82760300 |
| O | 5.98364400 | 2.28285900 | 1.58336500 |
| H | 5.91716800 | 3.69572800 | 3.09387000 |
| C | 5.42115400 | 2.89365400 | 2.53828400 |
| O | -4.18474800 | 2.48215600 | -3.03376900 |
| O | -5.94571600 | 2.39784900 | -1.77872000 |
| H | -5.82614600 | 3.69985600 | -3.38261500 |
| C | -5.36095200 | 2.91916800 | -2.77251600 |
| C | 1.56129900 | 4.65788400 | 0.75153700 |
| H | 2.22868900 | 3.83454600 | 1.01469600 |
| H | 1.51498300 | 4.75508100 | -0.33501200 |
| H | 1.95624600 | 5.58496300 | 1.17696200 |
| H | 0.56089600 | 4.48378200 | 1.15363600 |

## References

1. Zhang, X.; da Silva, I.; Godfrey, H. G. W.; Callear, S. K.; Sapchenko, S. A.; Cheng, Y.; Vitórica-Yrezábal, I.; Frogley, M. D.; Cinque, G.; Tang, C. C.; Giacobbe, C.; Dejoie, C.; Rudić, S.; Ramirez-Cuesta, A. J.; Denecke, M. A.; Yang, S.; Schröder, M. Confinement of Iodine Molecules into Triple-Helical Chains within Robust Metal−Organic Frameworks. *J. Am. Chem. Soc.* **2017**, *139*, 16289−16296.

2. Ai, L. H.; Li, L. L.; Zhang, C. H.; Fu, J.; Jiang, J. MIL-53(Fe): A Metal-Organic Framework with Intrinsic Peroxidase-Like Catalytic Activity for Colorimetric Biosensing. *Chem. - Eur. J.* **2013**, *19*, 15105−15108.

3. Horcajada, P.; Surblé, S.; Serre, C.; Hong, D.-Y.; Seo, Y.-K.; Chang, J.-S.; Grenèche, J.-M.; Margiolaki, I.; Férey, G. Synthesis and Catalytic Properties of MIL-100(Fe), an Iron(III) Carboxylate with Large Pores. *Chem. Commun.* **2007**, *38*, 2820−2822.

4. Zhu, K.; Liang, S.; Cui, X.; Huang, R.; Wan, N.; Hua, L.; Li, H.; Chen, H.; Zhao, Z.; Hou, G.; Li, M.; Jiang, Q.; Yu, L.; Deng, D. Highly Efficient Conversion of Methane to Formic Acid under Mild Conditions at ZSM-5-Confined Fe-Sites. *Nano Energy* **2021**, *82*, 105718.

5. Rowsell, J. L.; Yaghi, O. M. Effects of Functionalization, Catenation, and Variation of the Metal Oxide and Organic Linking Units on the Low-Pressure Hydrogen Adsorption Properties of Metal−Organic Frameworks. *J. Am. Chem. Soc.* **2006**, *128*, 1304−1315.

6. Xiang, S.; Zhou, W.; Gallegos, J. M.; Liu, Y.; Chen, B. Exceptionally High Acetylene Uptake in a Microporous Metal-Organic Framework with Open Metal Sites. *J. Am. Chem. Soc.* **2009**, *131*, 12415−12419.

7. Yang, S.; Sun, J.; Ramirez-Cuesta, A. J.; Callear, S. K.; David, W. I. F.; Anderson, D. P.; Newby, R.; Blake, A. J.; Parker, J. E.; Tang, C. C.; Schröder, M. Selectivity and Direct Visualization of Carbon Dioxide and Sulfur Dioxide in a Decorated Porous Host. *Nat. Chem.* **2012**, *4*, 887−894.

8. Xu, S.; Han, X.; Ma, Y.; Duong, T. D.; Lin, L.; Gibson, E. K.; Sheveleva, A.; Chansai, S.; Walton, A.; Ngo, D.; Frogley, M. D.; Tang, C. C.; Tuna, F.; McInnes, E. J. L.; Catlow, C. R. A.; Hardacre, C.; Yang, S.; Schröder, M. Catalytic Decomposition of $NO_2$ over a Copper-Decorated Metal−Organic Framework by Non-Thermal Plasma. *Cell Rep. Phys. Sci.* **2021**, *2*, 100349.

9. Ma, Y.; Han, X.; Xu, S.; Wang, Z.; Li, W.; da Silva, I.; Chansai, S.; Lee, D.; Zou, Y.; Nikiel, M.; Manuel, P.; Sheveleva, A. M.; Tuna, F.; McInnes, E. J. L.; Cheng, Y.; Rudić, S.; Ramirez-Cuesta, A. J.; Haigh, S. J.; Hardacre, C.; Schröder, M.; Yang, S. Atomically Dispersed Copper Sites in a Metal−Organic Framework for Reduction of Nitrogen Dioxide. *J. Am. Chem. Soc.* **2021**, *143*, 10977−10985.

10. Stere, C.; Chansai, S.; Gholami, R.; Wangkawong, K.; Singhania, A.; Goguet, A.; Inceesungvorn, B.; Hardacre, C. A Design of a Fixed Bed Plasma DRIFTS Cell for Studying the NTP-Assisted Heterogeneously Catalysed Reactions. *Catal. Sci. Technol.* **2020**, *10*, 1458−1466.

11. Yu, X.; Zholobenko,V. L.; Moldovan, S.; Hu, D.; Wu, D.; Ordomsky, V. V.; Khodakov, A. Y. Stoichiometric Methane Conversion to Ethane Using Photochemical Looping at Ambient Temperature. *Nat. Energy* **2020**, *5*, 511−519.
12. Wu, S.; Tan, X.; Lei, J.; Chen, H.; Wang, L.; Zhang, J. Ga-Doped and Pt-Loaded Porous $TiO_2$-$SiO_2$ for Photocatalytic Nonoxidative Coupling of Methane. *J. Am. Chem. Soc.* **2019**, *141*, 6592−6600.
13. Jiang, W.; Low, J.; Mao, K.; Duan, D.; Chen, S.; Liu, W.; Pao, C.-W.; Ma, J.; Sang, S.; Shu, C.; Zhan, X.; Qi, Z.; Zhang, H.; Liu, Z.; Wu, X.; Long, R.; Song, L.; Xiong, Y. Pd-Modified ZnO−Au Enabling Alkoxy Intermediates Formation and Dehydrogenation for Photocatalytic Conversion of Methane to Ethylene. *J. Am. Chem. Soc.* **2021**, *143*, 269−278.
14. Meng, L.; Chen, Z.; Ma, Z.; He, S.; Hou, Y.; Li, H.-H.; Yuan, R.; Huang, X.-H.; Wang, X.; Wang, X.; Long, J. Gold Plasmon-Induced Photocatalytic Dehydrogenative Coupling of Methane to Ethane on Polar Oxide Surfaces. *Energy Environ. Sci.* **2018**, *11*, 294−298.
15. Li, X.; Xie, J.; Rao, H.; Wang, C.; Tang, J. Platinum- and $CuO_x$-Decorated $TiO_2$ Photocatalyst for Oxidative Coupling of Methane to $C_2$ Hydrocarbons in a Flow Reactor. *Angew. Chem., Int. Ed.* **2020**, *59*, 19702−19707.
16. Lang, J.; Ma, Y.; Wu, X.; Jiang, Y.; Hu, Y. H. Highly Efficient Light-Driven Methane Coupling under Ambient Conditions Based on an Integrated Design of a Photocatalytic System. *Green Chem.* **2020**, *22*, 4669−4675.
17. Bajec, D.; Kostyniuk, A.; Pohar, A.; Likozar, B. Micro-Kinetics of Non-Oxidative Methane Coupling to Ethylene over Pt/$CeO_2$ Catalyst. *Chem. Eng. J.* **2020**, *396*, 125182.
18. Igenegbai, V. O.; Almallahi, R.; Meyer, R. J.; Linic, S. Oxidative Coupling of Methane Over Hybrid Membrane/Catalyst Active Centers: Chemical Requirements for Prolonged Lifetime. *ACS Energy Lett.* **2019**, *4*, 1465−1470.
19. Guo, X.; Fang, G.; Li, G.; Ma, H.; Fan, H.; Yu, L.; Ma, C.; Wu, X.; Deng, D.; Wei, M.; Tan, D.; Si, R.; Zhang, S.; Li, J.; Sun, L.; Tang, Z.; Pan, X.; Bao, X. Direct, Nonoxidative Conversion of Methane to Ethylene, Aromatics, and Hydrogen. *Science* **2014**, *344*, 616−619.
20. Taheraslani, M.; Gardeniers, H. Coupling of $CH_4$ to $C_2$ Hydrocarbons in a Packed Bed DBD Plasma Reactor: the Effect of Dielectric Constant and Porosity of the Packing. *Energies* **2020**, *13*, 468.
21. Lee, H.; Lee, D.-H.; Ha, J. M.; Kim, D. H. Plasma Assisted Oxidative Coupling of Methane (OCM) over Ag/$SiO_2$ and Subsequent Regeneration at Low Temperature. *Appl. Catal. A* **2018**, *557*, 39−45.
22. Kim, S.-S.; Lee, H.; Choi, J.-W.; Na, B.-K.; Song H. K. Methane Conversion to Higher Hydrocarbons in a Dielectric-Barrier Discharge Reactor with Pt/$\gamma$-$Al_2O_3$ Catalyst. *Catal. Commun.* **2007**, *8*, 1438−1442.

23. Li, L.; Li, G.-D.; Yan, C.; Mu, X.-Y.; Pan, X.-L.; Zou, X.-X.; Wang, K.-X.; Chen, J.-S. Efficient Sunlight-Driven Dehydrogenative Coupling of Methane to Ethane over a $Zn^{+}$-Modified Zeolite. *Angew. Chem., Int. Ed.* **2011**, *50*, 8299−8303.
24. Li, L.; Cai, Y.-Y.; Li, G.-D.; Mu, X.-Y.; Wang, K.-X.; Chen, J.-S. Synergistic Effect on the Photoactivation of the Methane C−H Bond over $Ga^{3+}$-Modified ETS-10. *Angew. Chem., Int. Ed.* **2012**, *51*, 4702−4706.
25. Yu, L.; Shao, Y.; Li, D. Direct Combination of Hydrogen Evolution from Water and Methane Conversion in a Photocatalytic System over Pt/$TiO_2$. *Appl. Catal. B* **2017**, *204*, 216−223.
26. Zhang, W.; Fu, C.; Low, J.; Duan, D.; Ma, J.; Jiang, W.; Chen, Y.; Liu, H.; Qi, Z.; Long, R.; Yao, Y.; Li, X.; Zhang, H.; Liu, Z.; Yang, J.; Zou, Z.; Xiong, Y. High-Performance Photocatalytic Nonoxidative Conversion of Methane to Ethane and Hydrogen by Heteroatoms-Engineered $TiO_2$. *Nat. Commun.* **2022**, *13*, 2806.
